# A North American stem turaco, and the complex biogeographic history of modern birds

**DOI:** 10.1186/s12862-018-1212-3

**Published:** 2018-06-25

**Authors:** Daniel J. Field, Allison Y. Hsiang

**Affiliations:** 1 Milner Centre for Evolution, Department of Biology and Biochemistry, University of Bath, Claverton Down, Bath, BA2 7AY UK; 2 Department of Geology and Geophysics, Yale University, 210 Whitney Avenue, New Haven, CT 06511 USA; 3 Department of Bioinformatics and Genetics, Swedish Museum of Natural History, 10405 Stockholm, Sweden

**Keywords:** Biogeography, Palaeontology, Turaco, Musophagidae, Phylogeny, Fossils, Gondwana, Dispersal, Otidimorphae, Macroevolution

## Abstract

**Background:**

Earth’s lower latitudes boast the majority of extant avian species-level and higher-order diversity, with many deeply diverging clades restricted to vestiges of Gondwana. However, palaeontological analyses reveal that many avian crown clades with restricted extant distributions had stem group relatives in very different parts of the world.

**Results:**

Our phylogenetic analyses support the enigmatic fossil bird *Foro panarium* Olson 1992 from the early Eocene (Wasatchian) of Wyoming as a stem turaco (Neornithes: Pan-Musophagidae), a clade that is presently endemic to sub-Saharan Africa. Our analyses offer the first well-supported evidence for a stem musophagid (and therefore a useful fossil calibration for avian molecular divergence analyses), and reveal surprising new information on the early morphology and biogeography of this clade. Total-clade Musophagidae is identified as a potential participant in dispersal via the recently proposed ‘North American Gateway’ during the Palaeogene, and new biogeographic analyses illustrate the importance of the fossil record in revealing the complex historical biogeography of crown birds across geological timescales.

**Conclusions:**

In the Palaeogene, total-clade Musophagidae was distributed well outside the range of crown Musophagidae in the present day. This observation is consistent with similar biogeographic observations for numerous other modern bird clades, illustrating shortcomings of historical biogeographic analyses that do not incorporate information from the avian fossil record.

**Electronic supplementary material:**

The online version of this article (10.1186/s12862-018-1212-3) contains supplementary material, which is available to authorized users.

## Background

Living birds are among the world’s most diverse and widely distributed tetrapods; they inhabit a myriad of different environments, and exhibit enormous disparity in their forms and lifestyles [1]. However, this striking diversity is not distributed evenly across the globe. Today, Earth’s lower latitudes support the majority of avian species-level and higher-order diversity, and many deeply diverging clades are restricted to present-day vestiges of Gondwana, including Africa, Australasia, and South America [2–4]. This common distributional pattern has prompted the proposal of a Gondwanan origin for living birds, a hypothesis that has been used to corroborate arguments for an ancient Mesozoic diversification of the avian crown group [5–7]. Such arguments, which have historically favored a vicariant Gondwanan origin for crown birds (Neornithes), have often ignored data gained from the Palaeogene fossil bird record, which has improved substantially in recent years thanks to new discoveries and diagnoses based on rigorous phylogenetic analyses [8]. In fact, phylogenetic hypotheses for many Northern Hemisphere Palaeogene bird fossils may cast doubt on the hypothesis of a Mesozoic Gondwanan origin of Neornithes, as many crown-clades with restricted extant distributions appear to have stem-group relatives in very different parts of the world [8–10]. For example, palaeontological analyses have suggested that taxa as diverse as total group seriemas (Cariamidae) [11–16], mousebirds (Coliidae) [17–22], and courols (Leptosomidae) [11, 23, 24] – all of which are currently restricted to formerly Gondwanan landmasses – have early stem-group representatives in the Palaeogene of the Northern Hemisphere. Furthermore, stem group representatives of some extant clades currently restricted to the New World (e.g.*,* hummingbirds, Trochilidae) were formerly distributed in the Old World [25–31], while the opposite is true for certain extant taxa endemic to the Old World (e.g.*,* the roller + ground roller clade, Coracioidea [32–35]). A recent attempt to address the biogeographic origin of Neornithes, incorporating the Cenozoic avian fossil record, suggests a Mesozoic origin in West Gondwana (comprising what is now South America, West Antarctica, and portions of East Antarctica), followed by subsequent expansion into North America via an early Palaeogene land mass linking South America and North America [4].

Earlier work on neornithine historical biogeography [3], which did not incorporate the Cenozoic avian fossil record, drew three major conclusions: 1) that Neornithes originated in the Southern Hemisphere; 2) that the distributions of major groups of crown birds were influenced by the breakup of Gondwana (which took place almost entirely in the Mesozoic); and 3), as a corollary of 2), that crown birds were not substantially affected by the end-Cretaceous mass extinction event. However, several recent divergence time analyses advocate a largely Cenozoic adaptive radiation of higher-level clades within the avian crown group [36, 37]. Furthermore, the Late Cretaceous fossil record suggests a devastating impact of the K-Pg mass extinction event on avian diversity ~ 66 million years ago (making it unlikely that many lineages of crown birds survived this extinction event) [38]. As a result, the origins of today’s pervasive ‘trans-Antarctic’ neornithine biogeographic distributions are in need of additional study [3]. Notably, analytical reconstructions of avian historical biogeography have only recently begun to incorporate the early fossil record of the avian crown group [4, 39, 40], placing a premium on robustly supported phylogenetic hypotheses for early crown bird fossils.

Despite the importance of addressing the phylogenetic position of Palaeogene fossil birds, numerous significant specimens are in need of reevaluation. One notable example is *Foro panarium* Olson 1992, from the Early Eocene (Wasatchian) Fossil Butte Member of the Green River Formation, Wyoming. The holotype and only known specimen of *F. panarium* (USNM 336261) is represented by a well-preserved, nearly complete, semi-articulated skeleton [41]. In the initial description of this specimen, Olson [41] noted several osteological features shared with extant Musophagidae (including similarities in the scleral ossicles, ectethmoid, and the presence and shape of the pectineal process of the pelvis), and cautiously referred *F. panarium* to Order Cuculiformes Wagler 1830. Despite noting that this grouping (comprising Opisthocomidae, Musophagidae, and Cuculidae) probably did not represent a monophyletic group, this taxonomic decision was made on the basis of greater perceived overall similarity between *F. panarium* and the Hoatzin (*Opisthocomus hoazin*), turacos (Musophagidae), and cuckoos (Cuculidae) than to any other clade of extant birds. Despite the thorough initial description of *F. panarium* [41], and the near-complete nature of the holotype, the position of *F. panarium* has not been reassessed in an explicitly phylogenetic context.

Here we show that new phylogenetic analyses support *Foro panarium* as a stem turaco (Pan-Musophagidae). We thereby extend the list of Palaeogene fossil birds exhibiting geographic distributions that are extremely dissimilar to those of their closest living relatives, as all extant turacos (comprising a clade of ~ 24 species) are endemic to sub-Saharan Africa [42]. Although the higher order phylogenetic placement of crown Musophagidae has historically been labile (e.g.*,* contrast [36, 37, 43–52]), several recent studies uphold a comparatively recent common ancestor of crown turacos and crown cuckoos (Cuculidae), possibly as part of a broader clade including crown bustards (Otididae) [36, 37, 46, 53].

## Methods

### Phylogenetic analysis

**Fig. 1:**
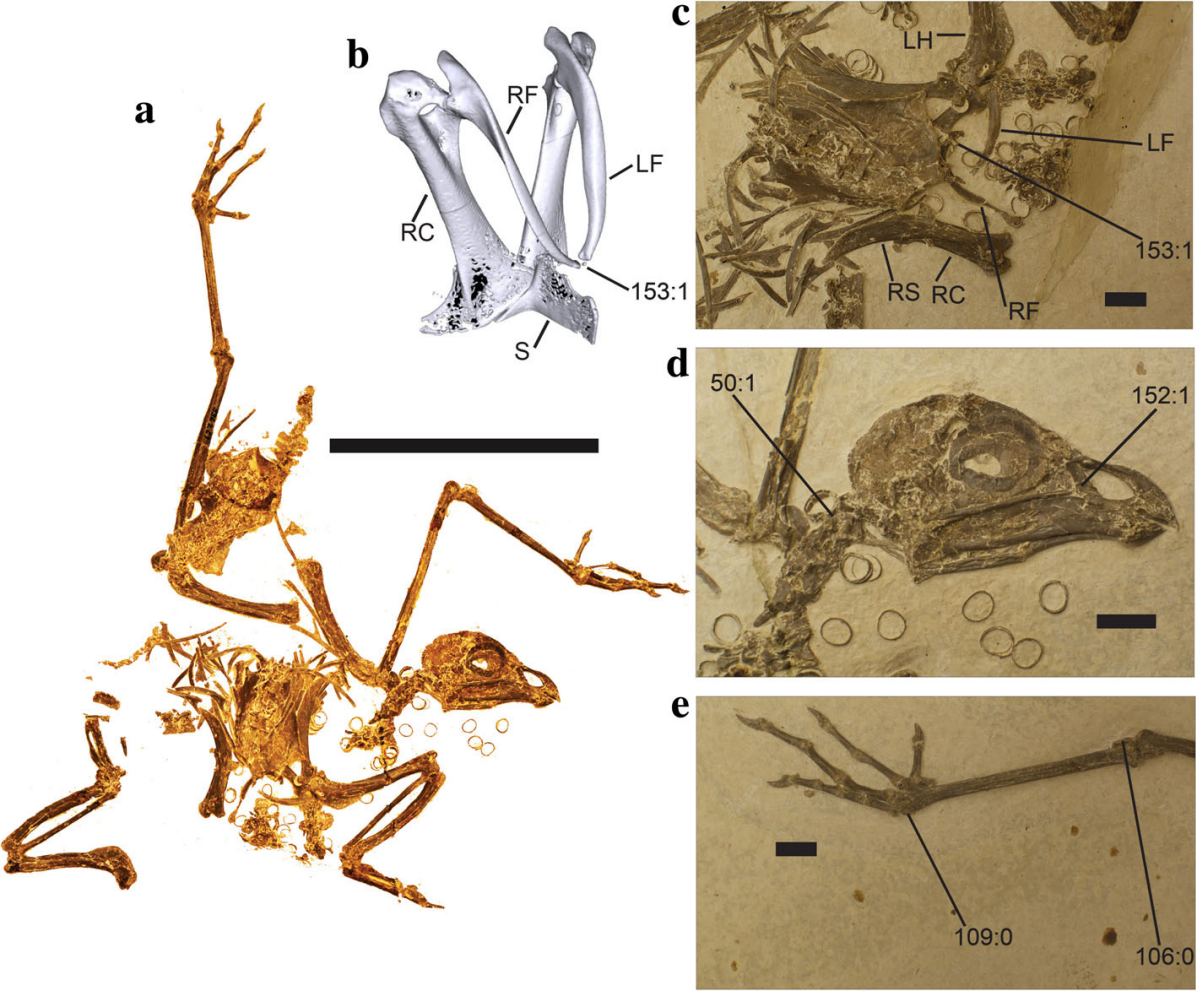
Skeletal morphology of total clade musophagids. (*a*) Complete skeleton of *Foro panarium* holotype USNM 336261. Scale bar equals 10 cm. (*b*) 3-dimensional CT rendering of the pectoral region of Ross’s Turaco (*Musophaga rossae*) GCO 1142 (Georgia College Ornithology, Georgia College and State University, Milledgeville, GA). LF – left ramus of furcula, RC – right coracoid, RF – right ramus of furcula, S – sternum. 153:1 denotes unfused midline of furcula, which optimizes as an unambiguous synapomorphy of a *Foro* + Musophagidae clade. (*c*) Pectoral region of *F*. *panarium*. LH – left humerus, RS – right scapula. (*d*) Cranial region of USNM 336261. 50:1 processus costales of axis absent. 152:1 bill short and stout with broad processus maxillaris of the os nasale. (*e*) Distal end of right leg of USNM 336261. 109:0 – trochlea metatarsi IV without large trochlea accessoria. 106:0 tendon of musculus flexor hallucis longus not enclosed in bony canal

We tested the phylogenetic position of *Foro panarium* (Fig. 1) by performing a suite of phylogenetic analyses under different topological constraints and optimality criteria. The holotype of *F. panarium* (which has never previously been included in a phylogenetic analysis) was coded from direct observation. Parsimony and Bayesian phylogenetic analyses were performed in PAUP* v.4.0b10 [54] and *MrBayes* v3.2.2 [55], respectively, with Palaeognathae specified as the outgroup. The character/taxon matrix (Additional file 1) consisted of 46 taxa and 153 morphological characters (of which 65 could be coded for *F. panarium*), and is based on a revised version of the dataset published by Mayr et al. [56] (itself a modification of Mayr and Clarke [50]). Two characters were newly added to the Mayr et al. dataset: 152. bill short and stout with broad processus maxillaris of the os nasale: no (0), yes (1); and 153. furcula unfused at midline: no (0), yes (1).

**Fig. 2:**
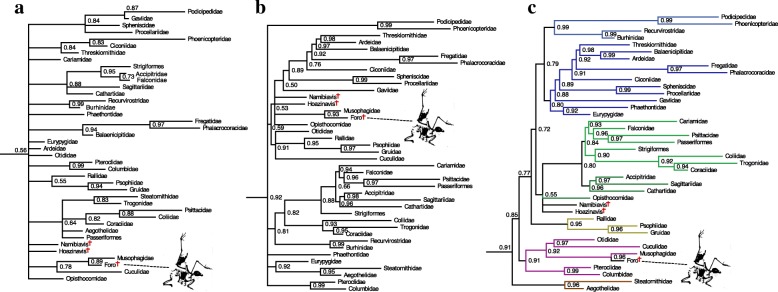
Bayesian phylogenetic analyses of a modified version of the Mayr et al. [56] dataset, conducted under a series of hierarchical topology constraints informed by the Hackett et al. [45] and Prum et al. [37] phylogenies. For simplicity, only Neoaves are shown; taxa with crosses are extinct. (*a*) Results of unconstrained analysis. (*b*) Results of analysis with extant taxa constrained to the 50% majority rule tree from Hackett et al. [45]. (*c*) Results of analysis with extant clades constrained to the topology from Prum et al. [37]. Colored branches in (*c*) reflect those used by Prum et al. [37]. Node support values are Bayesian posterior probabilities; *Foro panarium* is supported as the sister taxon to crown Musophagidae under all constraints and optimality criteria

Given pervasive incongruities between the morphological phylogenetic topology inferred by Mayr and Clarke [50] and recent phylogenomic analyses of neornithine interrelationships (e.g.*,* [36, 37, 45, 46, 57]), we analyzed the morphological matrix under a series of hierarchical topological constraints (‘scaffolds’ sensu Lee [58]) informed by the Hackett et al. [45] and Prum et al. [37] topologies. The same system of constraints was adhered to under parsimony and Bayesian optimality criteria. First, phylogenetic analyses were performed on the unconstrained morphological dataset (Fig. 2a). Subsequent analyses applied variations of the 50% ML majority-rule topology from Hackett et al. [45] as a topological scaffold (Fig. 2b, c, and d). These constrained analyses fixed the phylogenetic interrelationships of all taxa in the Hackett et al. [45] majority-rule topology, except those for which *Foro* was considered a potential fossil total-group representative by Olson [41] (i.e.*,* Opisthocomidae, Cuculidae, and Musophagidae). The first scaffold analysis (Backbone 1) did not fix the phylogenetic position of these three taxa, and subsequent analyses (Backbone 2–4) sequentially fixed the position of Opisthocomidae, Opisthocomidae + Cuculidae, and finally Opisthocomidae + Cuculidae + Musophagidae. Additionally, constrained analyses were performed following the recent phylogenomic topology of Prum et al. [37], applying the same methodology. Constraint trees are provided as separate Additional file 2.

Several osteological similarities between *F. panarium* and the Hoatzin (*Opisthocomus hoazin*) were noted in the original description of *F. panarium* [41]. Given the historical difficulty of identifying the Hoatzin’s extant sister taxon (the lineage leading to *O. hoazin* may represent the single longest branch in the neornithine tree of life [37]), and its strikingly unusual skeleton, an effort was made to shorten the phylogenetic branch leading to *O. hoazin* by including two stem opisthocomids in the phylogenetic analysis: *Hoazinavis lacustris* Mayr et al. 2011*,* from the late Oligocene/early Miocene of Brazil; and *Namibiavis senutae* Mayr 2014*,* from the late early Miocene of Namibia [56, 59]. The position of these fossils was left unconstrained in all phylogenetic analyses. Results of phylogenetic analyses are presented in Fig. 2.

For the parsimony analyses, heuristic searches were performed under tree-bisection-reconnection (TBR) branch swapping with 1000 random stepwise sequence addition replicates. Minimum branch lengths were set to collapse. Node support was calculated using bootstrap frequencies with 1000 bootstrap replicates and 10 random sequence addition replicates. Characters 55, 71, and 91 were treated as ordered (following the original Mayr and Clarke dataset [50]).

Bayesian phylogenetic analyses were run using *MrBayes* [55] on the CIPRES Science Gateway [60], using the *Mk* model of morphological evolution [61] with gamma-distributed rate variation and variable coding. All analyses were performed with two concurrent runs, sampling frequency of 1000, and four Metropolis-coupled chains (*T* **=** 0.1**)** for 10 million generations. Characters 55, 71, and 91 were again treated as ordered. Analyses were checked for convergence using standard *MrBayes* diagnostics (e.g.*,* PRSF< 0.01, mixing between chains > 20%) and *Tracer* (v1.5) [62] (e.g.*,* ESS > 200 for all estimated parameters). For all summary statistics, a relative burn-in of 25% was applied. Additional file 3: Table S1 provides the full character/taxon matrix. Although variations of the core anatomical dataset used to score *F*. *panarium* in this study have been subjected to numerous phylogenetic analyses under parsimony (e.g.*,* [50, 63–65]), to our knowledge, the present study represents the first time this matrix has been analyzed within a Bayesian phylogenetic framework.

### Historical biogeographic analyses

To obtain a time-scaled phylogeny, we used a majority-rule consensus tree identified by *Mesquite* [66] based on a sample of 1000 trees from the posterior distribution of Jetz et al. [67] (http://www.birdtree.org). This tree also applied the Hackett et al. [45] topology as a higher-order phylogenetic scaffold, allowing for direct comparisons with the constrained analyses described above. Following Cracraft [3], we restricted our sampling to crown neornithine family-level clades whose present-day biogeographic distributions are either exclusively Gondwanan or exclusively Laurasian.

The biogeographic history of birds in the Northern Hemisphere has been complex throughout the Cenozoic. In particular, the European fossil record indicates that several bird groups restricted to lower-latitudes today, including total group Struthionidae, Bucorvidae, and Opisthocomidae, may have migrated into higher latitude environments in the Northern Hemisphere during a period of warmer global temperatures around the Miocene climatic optimum ~ 15 MYA, after having arisen in lower-latitude settings [15, 68, 69]. In order to avoid conflating Palaeogene biogeographic patterns with subsequent overprinting of Northern Hemisphere avifaunas by dispersal from the tropics during the Miocene [69], we elected to restrict our fossil sampling to Palaeogene localities.

**Fig. 3:**
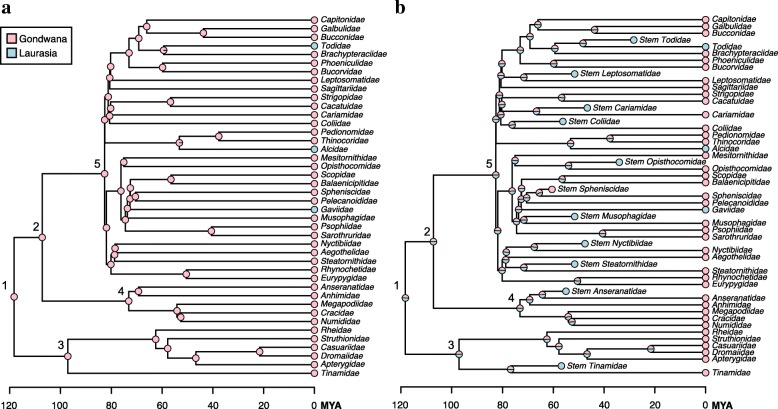
ML ancestral area reconstructions for extant family-level non-passerine taxa with modern distributions restricted to vestiges of Gondwana (pink) or Laurasia (blue) (trees taken from posterior distribution Jetz et al. [67] using the Hackett et al. [45] backbone). (*a*) Only extant taxa included. (*b*) Both extant taxa and well-constrained Paleogene fossils included. Although the extant-only analysis infers a Gondwanan neornithine ancestor with high probability (consistent with [3, 4]), including Paleogene fossils renders the root node reconstruction ambiguous. Alternative analyses with modified fossil representation and biogeographic scorings are presented in the supplement. Labeled nodes correspond to major neornithine clades: 1 = Neornithes, 2 = Neognathae, 3 = Palaeognathae, 4 = Galloanserae, 5 = Neoaves

To evaluate the complexity of avian biogeographic history in light of the Palaeogene bird fossil record, we performed two separate analytical reconstructions of the biogeographic origin of the avian crown clade using multiple techniques. These analyses were designed to test the capacity of fossils to illustrate profound fluctuations in avian biogeographic distributions that would otherwise be unknowable. The first was a quantitative approximation of the scenario put forth by Cracraft [3], incorporating every extant non-passerine clade traditionally ranked at the family level that exhibits an exclusively ‘Gondwanan’ or ‘Laurasian’ biogeographic distribution, and no fossils (Fig. 3a). Although certain clades (e.g.*,* Nyctibiidae) extend into southernmost North America, or even slightly beyond (e.g.*,* Cracidae), the northward extensions of their ranges are almost certainly attributable to contiguous dispersal following uplift of the Panamanian isthmus. Accordingly, these clades were scored as Gondwanan, as they were by Cracraft [3]. Other clades, such as the Podargidae (frogmouth) crown group, are found exclusively at lower latitudes, but extend from Australia into Southeast Asia (not a vestige of Gondwana). These clades were excluded from the Gondwanan-only coding scheme. It should be noted, however, that probable stem group podargids are known from the Palaeogene of both Europe and North America; as such, the Palaeogene distribution of the Podargidae total-group greatly exceeded the apparently relictual range occupied by the crown clade today (e.g.*,* [63, 70]). The same is true of several other extant pantropical groups not included in this analysis, including trogons (Trogoniformes) and parrots (Psittaciformes), both of which have extensive Northern Hemisphere total group fossil records [69].

The second analysis (Fig. 3b) builds upon the first by incorporating all well-supported extinct Palaeogene sisters to the extant clades in Fig. 3a. For a complete list of the fossil taxa included, see Table 1. We illustrate the ability of the avian fossil record to introduce important new biogeographic information by performing ancestral area reconstructions in a Bayesian framework using the *R* (v. 2.15.1) [71] package *phytools* (function *make.simmap*) [72], under the equal rates (ER) model with an estimated stationary prior distribution on the root, empirical rate matrix, and 500 simulation replicates. Extant and extinct taxa were coded as either ‘Gondwanan’ or ‘Laurasian’, and the results of these reconstructions are presented at the nodes of Fig. 3a and b.

**Table 1:** Fossil taxa included in biogeographic analysis, their closest crown group relatives, and stratigraphic age

Fossil Taxon	Crown sister	Age (Ma)	Locality	References
*Prefica nivea*	Steatornithidae	51.58	Green River Formation, USA	Nesbitt et al. 2011
*Sandcoleus copiosus*	Coliidae	56.22	Willwood Formation, USA	Houde and Olson 1992, Ksepka and Clarke 2009
*Palaeotodus* cf. *itardiensis*	Todidae	28.3	Escamps & Itardies, France	Mayr and Knopf 2007, Mayr 2009
*Foro panarium*	Musophagidae	51.58	Green River Formation, USA	Olson 1992, This study
*Lithornis celetius*	Tinamidae	56.8	Fort Union Formation, USA	Houde 1988, Nesbitt et al. in press
*Plesiocathartes wyomingensis*	Leptosomidae	51.58	Green River Formation, USA	Weidig 2006
*Dynamopterus tuberculata*	Cariamidae	46.6	Messel, Germany	Peters 1995, Mayr 2009, Mourer-Chauviré 2013
*Protoazin parisiensis*	Opisthocomidae	34	Romainville, France	Mayr and de Pietri 2014
*Waimanu manneringi*	Spheniscidae	60.5	Waipara Greensand, New Zealand	Slack et al. 2006
*Paraprefica kelleri*	Nyctibiidae	47.5	Messel, Germany	Nesbitt et al. 2011
*Anatalavis oxfordi*	Anseranatidae	55	London Clay, United Kingdom	Mayr 2008

In addition to the ‘simplistic’ biogeographic reconstruction described above, we performed biogeographical ancestral state reconstructions using RASP (v.3.2 build 20,160,719) [73] under two models: S-DIVA (Statistical-Dispersal Vicariance Analysis; [74]) and BayArea [75]. Both analyses were conducted using the phylogeny built from the full dataset containing both extant and fossil taxa, with Todidae coded as Laurasian (i.e., equivalent to the tree shown in Fig. 3b). As we use only a single phylogenetic tree and do not exclude any possible combined geographic ranges, our S-DIVA analysis is equivalent to using the standard DIVA model [76]. The maximum number of areas at each node was set to two, extinctions were allowed, and ancestral node ages were entered manually.

For the BayArea analysis, we represented the relative positions of Laurasia and Gondwana by using the centroid latitude/longitude coordinates of the two landmasses, as required by the BayArea algorithm. To do this, we used the program *GPlates* (v.1.5.0 build 16,091) [77] to reconstruct the tectonic plate positions of the two supercontinents at 118 MYA (the approximate root age of the Jetz et al. 2012 tree). The rotation model, coastline data, model gridmarks, global isochron data, continent-ocean boundary data, and spreading ridge data were sourced from Seton et al. [78]. The *GPlates* polygon digitization tool was used to trace approximate outlines of Laurasia and Gondwana by hand, resulting in 56 and 35 modern longitude-latitude coordinates, respectively. These coordinates were then exported as ESRI shapefiles, and the centroid coordinates of each polygonal area were then calculated using the *gCentroid* function in the *R* [71] package *rgdal* [79]. Because the polygon enclosing the Laurasian landmass overlaps with the North Pole, additional processing was needed to obtain its centroid due to the limitations of the *gCentroid* algorithm. Namely, the original longitude-latitude coordinates, which were specified under the WGS84 geographic coordinate system, were first converted to a planar coordinate reference system (CRS) via EPSG projection 3575 (North Pole LAEA [Lambert azimuthal equal-area] Europe) using the *rgdal* function *spTransform*. The centroid was then calculated using the converted planar coordinates using the *gCentroid* function. Finally, the planar centroid coordinates were reconverted into longitude-latitude coordinates under the WGS84 CRS. The final coordinates were 79.65 lat and 27.97 long for Laurasia and − 43.32 lat and 9.41 long for Gondwana. The BayArea analysis was run using these coordinates for 50 million generations, sampling every 1000 generations, using default parameter and prior settings. A 25% burn-in of 12.5 million generations was implemented after the run before summarization of results.

Recently, the use of fossil tip-dating [80] to investigate the influence of fossils on ancestral state reconstructions has increased, allowing for more accurate reconstructions (e.g.*,* [81]). However, due to the lack of a sufficiently taxonomically-inclusive anatomical phylogenetic dataset for the avian crown, a ‘total-evidence’ phylogenetic analysis that explicitly incorporates nucleotide sequences and anatomical data across crown bird diversity, along with fossil data from Palaeogene representatives, was beyond the scope of this investigation (although such an analysis has recently been applied to the avian subclade Galloanserae [82]). Instead, we incorporated Palaeogene fossils into the Jetz et al. [67] time-calibrated consensus tree by grafting them into their phylogenetic positions as inferred by independent phylogenetic analyses (Table 1). The ages of the fossils grafted into the consensus tree followed the age of the earliest well-supported representative of the fossil species (Table 1). These age estimates follow best practices for justifying minimum age constraints [83]; thus, we apply the youngest possible age for each fossil, inclusive of error. The node subtending each grafted fossil and its extant sister clade was placed 20 million years prior to the age of the fossil, except for the nodes within total-clade Spheniscidae and Anseranatidae, because this would conflict with the age for the node subtending crown Spheniscidae and Anseranatidae and their extant sisters from the Jetz et al. [67] analysis. For these clades, a five million-year offset was applied instead. See Additional file 2 for details regarding alternative biogeographic parameterizations for taxa with biogeographic distributions that were challenging to characterize.

### Morphometric analyses

**Fig. 4:**
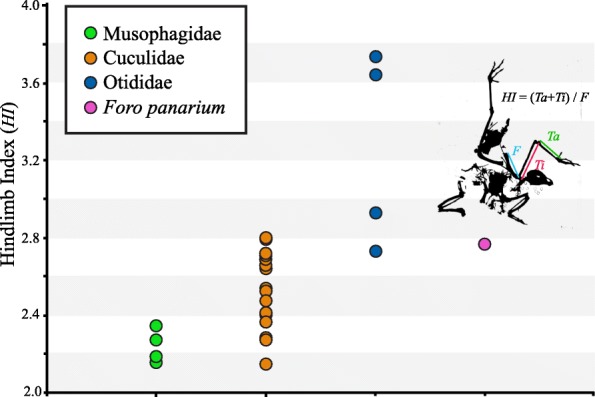
Comparison of hindlimb proportions among the major clades of Otidimorphae. Hindlimb Index is computed as the ratio between the sum of tarsometatarsus + tibiotarsus length, and femur length (illustrated on a silhouette of holotype skeleton of *Foro panarium*). Unlike crown group Musophagidae, *F. panarium* exhibited long, gracile legs more similar to a small bustard or the neomorphine cuckoo *Dromococcyx phasianellus.* The lowest Hindlimb Index value overall is exhibited by the putative stem cuculid, *Eocuculus* cf. *cherpinae*

Digital calipers sensitive to 0.01 mm were used to measure the total length of the femur, tibiotarsus, and tarsometatarsus for adult representatives of Otidimorphae (Musophagidae, Cuculidae, and Otididae). Measurements are presented in Additional file 4: Table S2 and plotted in Fig. 4. In total, six species of Musophagidae were examined, representing every major musophagid subclade. Twenty-eight species of Cuculidae were examined from across the extant diversity of cuckoos, and four species of Otididae were measured. Measurements from extant taxa were compared to measurements from *Foro panarium* presented in Olson [41] and to the possible stem cuculid *Eocuculus cherpinae* [84, 85]. A hindlimb index was computed as the ratio between the sum of tibiotarsus and tarsometatarsus length, and femur length. Results of morphometric comparisons are displayed in Fig. 4.

## Results

### Phylogenetic analyses

Despite exhibiting an enigmatic combination of osteological features not seen in any extant birds [41], *Foro panarium* was supported in our analyses as the extinct sister to crown Musophagidae under all optimality criteria and topological constraints (Fig. 2). In our Bayesian analyses, posterior probabilities for the exclusive *Foro*-Musophagidae clade ranged from 0.80 (Hackett Backbone with cuckoos, hoatzin, and turacos unconstrained) to 0.96 (Prum Constraint). Under parsimony, the *Foro*-Musophagidae node was supported with bootstrap scores ranging from 0.57 (Hackett Backbone with cuckoos, hoatzin, and turacos unconstrained) to 0.95 (Hackett Backbone with all extant taxa constrained). In the unconstrained parsimony analyses, the most parsimonious tree (which resolved *Foro* as sister to Musophagidae) was found to be 215 steps; 217 were necessary to resolve *Foro* as sister to Cuculidae, and 220 were necessary to resolve *Foro* as sister to Opisthocomidae.

We found substantial incongruence between our unconstrained morphological analyses and recently proposed genetic sequence-based hypotheses for neornithine interrelationships (e.g.*,* [36, 37, 45]) under both parsimony and Bayesian optimality criteria, underscoring the extant avian radiation as an exemplar of discordance among alternative phylogenetic datasets [86, 87]. Although considerable progress has been made toward resolving the deepest nodes within the neoavian radiation, complete consilience has been elusive even among recent next-generation phylogenomic datasets [36, 37], perhaps as a result of widespread incomplete lineage sorting during the early stages of the neoavian radiation [88], or long-branch attraction artifacts in datasets with sparse taxonomic samples [37]. From an anatomical standpoint, achieving congruence between phenotypic and genotypic analyses of neornithine interrelationships demands a renewed focus on expanding and refining existing neornithine character/taxon datasets, and additional effort to integrate phenotypic and genotypic data in phylogenetic studies [80–82, 89, 90].

*Foro panarium* exhibits a furcula unfused at its midline (Character 153:1), which is recovered as an unambiguously optimized synapomorphy of a *Foro* + Musophagidae clade. Although the majority of the left furcular ramus is separated from the right ramus and rotated perpendicular to its original position (Fig. 1), the medial portion of the left ramus is in its original orientation. Together with the complete and unbroken right furcular ramus, the medial portion of the left ramus bounds an unfused midline symphysis. Together, the medial and lateral components of the left furcular ramus match the length and shape of the intact right ramus. Monophyly of crown Musophagidae to the exclusion of *F*. *panarium* is supported by several character states, including: a largely ossified septum nasale (character 8:1); a pygostyle perforated by a foramen at its caudoventral end (character 61:0); an extremitas omalis of the furcula with strongly developed, laterally protruding facies articularis acrocoracoidea (character 62:1); and crista deltopectoralis of humerus extending distal to crista bicipitalis and proximodorsal portion of bone with sigmoidally curved margin (character 151:1). *F. panarium* exhibits the same states for these four characters that bustards (Otididae) do, which presumably reflects the plesiomorphic condition for Otidimorphae.

Although character state optimization varied across our alternative phylogenetic analyses, character states consistently optimizing as unambiguous local synapomorphies of a *Foro panarium* + Musophagidae clade included 93:0 (large tubercula praeacetabularia of the pelvis); 86:1 (carpometacarpus, proximal end of os metacarpale minus dorsoventally wide and strongly deflected ventrally); 87:1 (os carpi ulnare with crus longum greatly abbreviated); 152:1 (bill short and stout with broad processus maxillaris of the os nasale); and 153:1 (furcula unfused at midline). Character state 87:1 was posited as a potential synapomorphy of a Musophagidae + Cuculidae clade by Hughes [53]—accordingly, this character state did not optimize as an unambiguous *F. panarium* + Musophagidae synapomorphy in analyses imposing backbone constraints where Musophagidae and Cuculidae are closely related (e.g. [37]), and may in fact represent a synapomorphy for Otidimorphae more generally.

Despite the inclusion of the branch-shortening stem hoatzins *Hoazinavis lacustris* and *Namibiavis senutae* [56, 59], an exclusive *Foro* + Opisthocomidae clade was never recovered. In our Bayesian analyses, *N. senutae* and *H. lacustris* never formed an exclusive clade with crown Opisthocomidae under majority rules consensus (instead emerging in a polytomy with *Opisthocomus*), likely due to missing data arising from the incomplete nature of those fossil specimens. Osteological similarities between *F. panarium* and *Opisthocomus hoazin*, which mostly relate to the overall shape of the skull and mandible (including the ‘short, hook-like retroarticular process’ [41]), are therefore best interpreted as the result of convergence.

### Ancestral area reconstructions

Our historical biogeographic reconstructions revealed marked differences between analyses including and excluding Palaeogene fossil birds. In the extant-only biogeographic reconstruction (Fig. 3a), the hypothesis that pervasive ‘trans-Antarctic’ biogeographic patterns across crown birds indicate a Southern Hemisphere origin for Neornithes was strongly supported (*pro* [3]). Indeed, the maximum likelihood ancestral area reconstruction for the neornithine root is inferred to have been Gondwanan with 100% probability. However, inclusion of Palaeogene bird fossils obfuscates this inference (Fig. 3b). All nine variations of the extant+fossil dataset yield ambiguous reconstructions, with the probability of an exclusively Gondwanan root node varying between 45.2 and 52.2% (Additional file 5: Figure S1, *contra* [3]). This analysis illustrates the unique potential of the fossil record to illustrate where representatives of major avian subclades were formerly distributed, and underscores the profound historical fluctuations in avian biogeography revealed by the fossil record.

**Fig. 5:**
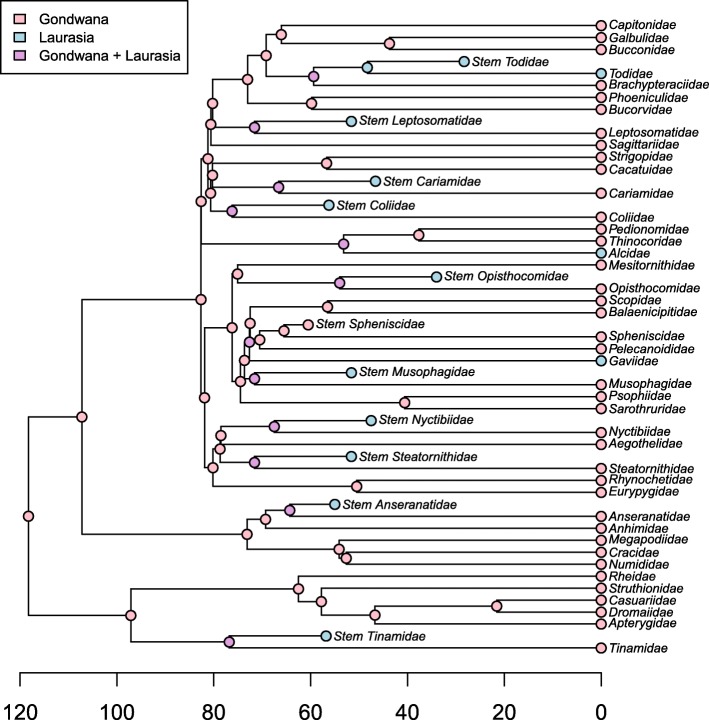
Results of S-DIVA analysis of the combined extant+fossil dataset, yielding a 100% Gondwanan reconstruction for the avian root. This method does not incorporate branch lengths—a potential limitation underscoring the speciously confident statistical reconstruction

**Fig. 6:**
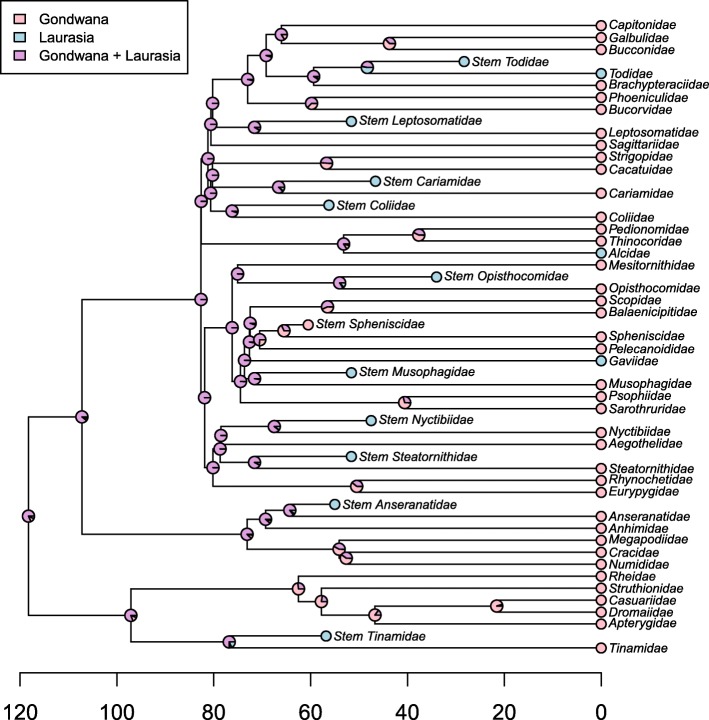
Results of BayArea analysis of the combined extant+fossil dataset, yielding a more ambiguous result. The root node is reconstructed as 81.7% Laurasian/Gondwanan, 11.3% Gondwanan, and 7% Laurasian

The results of our S-DIVA analysis are shown in Fig. 5. This method reconstructs the root as being 100% Gondwanan despite the inclusion of Laurasian fossils, similar to our naïve likelihood extant-taxa-only results. Under the simple DIVA model, branch lengths are ignored; we believe that this limitation of the method explains the simplistic 100% Gondwanan reconstruction, due to the ubiquity of Gondwanan distributions among the extant taxa in our analysis. In contrast, the BayArea analysis reconstructs a far more ambiguous result, with the root node reconstructed as 81.69% Laurasian/Gondwanan, 11.33% Gondwanan, and 6.98% Laurasian (Fig. 6).

### Morphometric analyses

Hindlimb ratios (HR) for extant musophagids (which are arboreal) are uniformly low, with the highest measured musophagid ratio exhibited by the White-bellied Go-away Bird, *Corythaixoides leucogaster* (HR = 2.32) (Fig. 4; Additional file 4: Table S2). Musophagid hindlimb ratios are exceeded by most Cuculidae, although the lowest overall hindlimb ratio was exhibited by the generally arboreal Common Cuckoo, *Cuculus canorus* (HR = 2.13). The exclusively terrestrial Otididae exhibit uniformly high hindlimb ratios, with exceptionally high values seen in *Ardeotis* (*A. arabs,* HR *=* 3.63; *A. kori,* HR *=* 3.53). *Foro panarium* exhibits a hindlimb ratio (HR = 2.77) intermediate between that of the Little Bustard (*Tetrax tetrax*, HR = 2.66) and the Great Bustard (*Otis tarda*, = 2.85), which also closely corresponds to ratios exhibited by some (terrestrial) neomorphine ground cuckoos, including *Dromococcyx phasianellus* (HR = 2.78) and *Geococcyx californianus* (HR = 2.70). The hindlimb ratio of *F*. *panarium* greatly exceeds those of any extant musophagids, although the femur length (54.1 mm) falls within the range of variation exhibited by crown turacos, suggesting a live body mass between 636 g–714 g [91]. This estimated body mass falls between the mean body size of the large extant turacos *Crinifer zonurus* and *Corythaeola cristata*, and within the known range of variation for small extant bustards such as *Eupodotis ruficrista* [92].

## Discussion and conclusions

### The evolutionary history of total group turacos (pan-Musophagidae)

Crown turacos (Musophagidae, Musophagiformes) comprise ~ 24 traditionally recognized species, many of which exhibit vivid plumage coloration conferred by unique porphyrin pigments [42]. Until recently, the phylogenetic position of crown turacos had been among the most recalcitrant phylogenetic problems in neornithine systematics, with turacos having been alternatively allied with a number of distantly related clades (e.g.*,* cuckoos [93, 94], the hoatzin [95], mousebirds [43], and waterbirds [45]). Recently, however, the phylogenetic position of turacos within Neoaves has begun to come into focus, with congruent results emerging from next-generation phylogenomic datasets supporting a close relationship between turacos, bustards (Otididae), and cuckoos (Cuculidae) [36, 37, 46] (although McCormack et al. [46] did not sample Cuculidae). Both McCormack et al. [46] and Jarvis et al. [36] inferred a sister-group relationship between turacos and bustards, whereas Prum et al. [37] inferred a sister group relationship between turacos and a bustard + cuckoo clade. The most exclusive clade uniting Musophagidae, Cuculidae, and Otididae, with variable ingroup interrelationships, has been named Otidimorphae [36, 37].

The known fossil record of early turacos is sparse. Although molecular divergence dating analyses conflict markedly on the estimated antiquity of the turaco total group (e.g.*,* contrast Mesozoic estimates from Jetz et al. [67] and Palaeogene estimates from Prum et al. [37]), no convincing total group turaco fossils have previously been identified in sediments older than the early Oligocene [8, 96, 97]. Other putative Oligocene records of turacos [98] have been reinterpreted as belonging to other clades [8]. Our inference that *Foro panarium* represents an early Eocene stem turaco provides a well-supported calibration point for divergence time analyses as both the earliest representative of the turaco total group and the earliest fossil representative of Otidimorphae, thereby filling a major gap in the Cenozoic neornithine fossil record.

Assuming a post-Cretaceous radiation of crown Neoaves [36–38, 44, 99, 100], an early Eocene (~ 52.1MYA) stem turaco might be expected to exhibit a mix of crown turaco apomorphies as well as plesiomorphies that have been overwritten in extant Musophagidae. Indeed, *Foro panarium* appears intermediate, possessing some crown turaco apomorphies (e.g.*,* unfused furcula), while still exhibiting numerous osteological plesiomorphies (e.g.*,* unossified septum nasale; lack of prominent quill knobs on ulna).

Among the most surprising features of the skeleton of *Foro panarium* is the presence of long, gracile legs [41]. Extant turacos are highly arboreal, and exhibit fairly short legs (Fig. 4; Additional file 4: Table S2). The emerging hypothesis that turacos share a recent common ancestor with bustards (large-bodied, terrestrial birds with long legs) raises questions regarding the evolution of divergent hindlimb proportions in extant otidimorphs [36, 37]. Among crown group Otidimorphae, the hindlimb proportions of *F. panarium* are much more similar to those of bustards and certain New World ground cuckoos than they are to extant turacos (Fig. 4). The elongated hindlimbs of *F. panarium* may therefore approximate the ancestral condition for Otidimorphae [101]. The shortened hindlimbs of many cuckoos and turacos may have arisen either on their shared stem to the exclusion of bustards (as advocated by the Jarvis et al. [36] TENT topology), or independently along both the crownward portion of the turaco stem and the cuckoo stem (following the Prum et al. [37] topology). Unfortunately, the early evolutionary history of Cuculidae is poorly understood, with no well-supported stem group representatives [8]. As a result, the total group cuckoo fossil record may have little to contribute to the discussion of the pattern and timing of otidimorph hindlimb reduction – and the evolution of arboreal habits – at present. However, the late Eocene-early Oligocene taxon *Eocuculus cherpinae* Chandler 1999 (known from the Florissant Beds of Colorado and the Lubéron area of France) may represent a stem cuculid, although this hypothesis awaits further testing on the basis of phylogenetic analysis and the discovery of additional fossils [84, 85, 102]. Based on measurements of the holotype [85], the hindlimb index of *E. cherpinae* is considerably lower than any extant otidimorph (Fig. 4; Additional file 4: Table S2); if this taxon does in fact represent a stem cuculid, it may suggest that cuckoos arose from taxa with extremely short hindlimbs. However, a clade comprising the relatively long-legged ground cuckoos (Neomorphinae), together with the new world Crotophaginae, represents the extant sister taxon to all other extant cuckoos [103]. The phylogenetic position of Neomorphinae is consistent with a long-legged plesiomorphic condition for Otidimorphae, which would suggest that the short legs of *E. cherpinae* are autapomorphic (if *E. cherpinae* is indeed a stem cuckoo). Hindlimb proportions of *F. panarium* and other otidimorphs, in combination with exclusively terrestrial habits in bustards and widespread terrestrial ecologies within the extant cuckoo radiation, suggest that turacos may be descended from ground-dwelling antecedents, and that the arboreal habits of extant turacos arose relatively recently in their evolutionary history [101].

Claramunt and Cracraft [4] infer the probability of a South American vs. African origin for Pan-Musophagidae as equally parsimonious. If a South American origin for total-clade turacos is accurate, then the presence of an early stem turaco in the Eocene of North America is consistent with their interpretation of an early Cenozoic dispersal event from South America through North America (the ‘North American Gateway’ hypothesis). This scenario is congruent with prior inferences of North American origins for the (presently) Old World clades Coliidae and Leptosomidae, as well as the cosmopolitan Coraciiformes, corroborating the argument that North America played a pivotal role in the early evolutionary history of clades that subsequently radiated in the Old World [4].

Given the striking similarity of North American and European avifaunas in the early Eocene [32, 104, 105], it would be somewhat surprising if stem group turacos are not eventually recovered from Messel, or other early Cenozoic European localities. Europe was connected to North America across the Greenland-Scotland ridge in the early Eocene [106–108], which has been causally attributed to similarities in mammalian, squamate, and avian faunas from that time [109–111]. However, given that only one specimen referable to *Foro panarium* has yet been identified, it is possible that early stem turacos were comparatively rare in habitats surrounding lakes such as those of the Green River system. If so, they may have been similarly uncommon in the lakeside habitats surrounding Messel [112]. Numerous avian taxa from the Palaeogene of the Northern Hemisphere, whose present-day distributions are restricted to the Afrotropical zoogeographic realm (e.g.*,* Coliiformes), are known to have persisted in Europe until the Miocene [69]. Therefore, as-yet undetected early stem group musophagids may have survived longer in Europe than they did in North America (if they were present in Europe in the first place), and may be expected to be recovered in stratigraphically younger Palaeogene European localities.

### Implications for molecular divergence time analyses

The early Eocene Fossil Butte Member of the Green River Formation has been the subject of detailed dating analyses. Smith et al. [113] reported a radiometric date of 51.97 ± 0.16 Ma from a K-feldspar tuff just above the middle unit of the Fossil Butte Member [86]. As a result, *Foro panarium* easily represents the oldest representative of Otidimorphae yet known. Although undescribed fossils purportedly belonging to Otididae have been recovered from the Oligocene of Kazakhstan [114] and from an unknown horizon within the Oligocene Quercy fissure fillings of France [16], no pre-Oligocene remains of Otididae have yet been reported. Similarly, although extant Cuculidae are distributed on every continent except Antarctica [115], potential fossil representatives of total-clade Cuculidae are unknown from sediments older than the late Eocene Florissant Fossil Beds of Colorado, the roughly contemporaneous Cypress Hills Formation of Saskatchewan, and the early Oligocene Pichovet locality in France [8, 84, 85, 102, 116].

Our inferred phylogenetic position for *Foro panarium* as sister to Musophagidae hints not only at the antiquity of the musophagid total group, but also indicates that additional phylogenetic divergences within Otidimorphae had taken place by the early Eocene. If Musophagidae represents the extant sister taxon to a Cuculidae + Otididae clade (sensu [37]), then at least the stem group of this latter clade would have been present in the early Eocene. If, instead, Cuculidae is sister to a Musophagidae + Otididae clade (sensu [36]), then stem representatives of all three of these extant clades must have been present in the early Eocene.

*Foro panarium* therefore represents a valuable calibration point for neornithine molecular divergence dating analyses. Otidimorphae are among the deepest diverging clades within living birds (diverging near the base of Neoaves; [36, 37]). Thus, the presence of a stem musophagid in the early Eocene of North America has important implications for understanding the tempo of the extant neornithine radiation—one of the most contentious topics in contemporary ornithology [38, 117–122]. Recent summaries of useful criteria for well-justified fossil calibrations (e.g.*,* [83, 123–125]) identify six desirable features for a potential calibrating fossil; *F. panarium* satisfies all of these criteria, and therefore should help shed new light on the tempo and mode of the extant avian radiation.

### Neornithine historical biogeography and the importance of fossils

The importance of incorporating fossils into macroevolutionary analyses has been well documented. Including fossils can meaningfully influence phylogenetic analyses (e.g.*,* [81, 126–130]), ancestral state reconstructions (e.g.*,* [81, 120, 131]), and models of trait evolution (e.g.*,* [132, 133]). Similarly, synthesizing biogeographic data for extant clades and their fossil relatives has potential to provide a more holistic understanding of the dynamics of geographic range evolution than do studies focused exclusively on either the current distributions of clades or those of their fossil antecedents [134–137]. Indeed, the Palaeogene record of fossil birds has been discussed at length with respect to the historical biogeography of crown birds [8–10, 15, 32, 104, 105, 138, 139]. However, the results of these studies have rarely been incorporated into historical biogeographic analyses of living birds groups (however, see [4]). As a result, the Palaeogene fossil record of birds has often been dismissed for having been inadequately examined from a phylogenetic perspective (e.g.*,* [3]). Although much of the early work on Palaeogene bird fossils was conducted outside of an explicitly phylogenetic paradigm (including the initial description of *Foro panarium* [41]), a host of Palaeogene birds have been subjected to phylogenetic analyses over the last 20 years, providing strong evidence for the presence of the stem lineages of many higher clades (‘Orders’ under the traditional Linnean hierarchy) in the Palaeogene, often well outside the geographical limits of their respective crown clades [8].

The present study represents one of the first efforts to explicitly incorporate the Palaeogene neornithine fossil record into analytical reconstructions of higher-order avian historical biogeography. The results underscore the importance of the fossil record for informing our understanding of both the pattern by which crown neornithine biogeography has evolved and the timing of the extant neornithine radiation. Cracraft [3] identified numerous examples of apparent trans-Antarctic distribution patterns across the neornithine tree of life – that is, avian higher clades restricted to landmasses that were formerly part of the Gondwanan supercontinent (i.e.*,* Africa, Madagascar, Antarctica, Asia, Australia/New Zealand, and South America). Indeed, an ancestral area reconstruction based only on the current geographic distributions of these clades recovers a maximally-supported result in which both the most recent common ancestor of all living birds, and most internal nodes, favor an origin somewhere within Gondwana or its derivatives (Fig. 3a). This result has been interpreted to suggest that vicariance related to the breakup of the Gondwanan supercontinent, which was largely complete by the end of the Mesozoic era, was responsible for establishing this striking biogeographic pattern [3]. Because this biogeographic scenario necessarily invokes numerous neornithine divergences deep in the Cretaceous, this scenario has also been taken as support for a major Mesozoic radiation of crown birds [3]. Despite apparent corroboration of this temporal scenario by numerous molecular divergence time analyses [67, 140–142], the hypothesis of a major radiation of the neornithine crown in the Mesozoic has elicited mounting criticism in recent years. Recent molecular divergence dating analyses have begun to corroborate a rapid post-Cretaceous radiation of the major groups of crown birds [36, 37, 120–122]. If the results of these more recent analyses reflect the approximate age of the avian crown, any model invoking Mesozoic Gondwanan vicariance as a driver of crown avian biogeographic patterns can be soundly rejected.

Large-scale analytical reconstructions of the biogeographic origin of crown birds favor a West Gondwanan origin of Neornithes (comprised of the landmasses now belonging to South America and portions of Antarctica), a hypothesis that is robust to the inclusion of numerous Palaeogene fossils from the Northern Hemisphere [4]. Although including Palaeogene fossils in our biogeographic analysis rendered the ‘Gondwanan vs. Laurasian’ reconstruction ambiguous (Fig. 3), that result is not inconsistent with the West Gondwanan origin of crown birds advocated by Claramunt and Cracraft [4]. Regardless, it is clear that the Palaeogene fossil bird record from the Northern Hemisphere will be instrumental for unraveling the biogeographic history of Neornithes. Indeed, the presence of *Foro panarium* in the early Palaeogene of North America, and its identification as a stem turaco, is consistent with the ‘North American Gateway’ hypothesis – the idea that, after initially diverging in West Gondwana, North America served as a stepping stone for many avian clades on their way to colonizing the Old World via high latitude land bridges [4]. The continued identification of Laurasian fossils belonging to the early stem lineages of clades presently restricted to the Southern Hemisphere, such as *F. panarium*, may fundamentally alter our understanding of the early biogeographic origins of living bird clades. Continued efforts to assess the phylogenetic affinities of bird fossils from the Northern and Southern Hemispheres will be a crucial step towards fully understanding the biogeographic and temporal origins of modern bird diversity.

The ambiguity of the BayArea result mirrors that obtained from our naïve likelihood method with fossils included, and underscores the important influence of branch length information in historical biogeographic reconstructions (and comparative analyses more broadly). Although most of the extant taxa in our analyses are found on modern landmasses that comprised the Gondwanan paleo-supercontinent, most stem fossil representatives of these groups are found instead on Laurasia, and are subtended by relatively short branches – particularly in comparison to the length of subtending branches leading to extant taxa, along which there is comparatively ample opportunity for biogeographic events such as dispersal and vicariance to occur. In conjunction with our naïve likelihood analyses, the results of our explicitly biogeographic reconstructions of the distribution of early birds provide an important illustration of the influence of fossils in such analyses. When informative branch length data are incorporated, the fossil record provides indispensable data on evolutionary and biogeographic history, leading to reconstructions that may be unexpected when one considers extant data alone. This is true even when analyses containing only extant data result in seemingly unambiguous and unequivocal reconstructions (e.g., a 100% Gondwanan reconstruction for the root node). Indeed, our results serve as a cautionary warning against overreliance on extant data and high statistical support values when studying evolutionary processes that are fundamentally historical in nature.

## Additional files

Additional file 1: Additional information on materials and methods. (DOCX 142 kb)

Additional file 2: Nexus file for phylogenetic analysis. (ZIP 7 kb)

Additional file 3: **Table S1.** Character/taxon matrix used for the phylogenetic analyses. Character descriptions follow Mayr 2011 for characters 6, 15, 22, 31, 32, 44, 57, 58, 65, 67, 85, 94, 100, 105, 106, 149, 150, and 151. For the remaining characters, descriptions follow Mayr & Clarke 2003, except newly added characters 152 and 153 as described in the main text. (PDF 123 kb)

Additional file 4: **Table S2.** Hindlimb measurements for fossil and recent Otidimorphae. (PDF 70 kb)

Additional file 5: **Figure S1.** Relative likelihood of a Gondwanan vs. Laurasian avian common ancestor based on alternative parameterization of the historical biogeographic analyses. (PNG 211 kb)

## Abbreviations

K-Pg: Cretaceous-palaeogene
MYA: Million years ago
