# Supplementary material for: A North American stem turaco, and the complex biogeographic history of modern birds
Source: BMC Evol Biol. 2018 Jun 25;18:102. doi: 10.1186/s12862-018-1212-3 (PMC6016133; doi:10.1186/s12862-018-1212-3)
Supplement: Supplementary file 1 — Additional information on materials and methods. (DOCX 142 kb) [file 12862_2018_1212_MOESM1_ESM.docx]

**Electronic Supplementary Material**

**Supplementary Materials and Methods**

Phylogenetic analysis

We tested the phylogenetic position of *Foro panarium* by performing a suite of phylogenetic analyses under different optimality criteria. Parsimony and Bayesian phylogenetic analyses were performed in *PAUP** (v.4.0b10) [1] and *MrBayes* v3.2.2 [2], respectively. The character/taxon matrix consisted of 46 taxa and 153 morphological characters (of which 65 could be coded for *Foro*), and is based on a revised version of the dataset published by Mayr *et al*. [3] (itself a modified version of that originally published by Mayr and Clarke [4]). Two characters were newly added to the Mayr *et al.* [5] dataset: 152. Bill short and stout with heavy, complete lateral nasal bar broadly fused to the ventral bar of premaxilla: no (0), yes (1); and 153. Furcula unfused at midline: no (0), yes (1).

For the parsimony analyses, Palaeognathae was specified as the outgroup, and heuristic searches were conducted using tree-bisection-reconnection (TBR) branch swapping with 1,000 replicates of random stepwise sequence addition. Minimum branch lengths were set to collapse. Support for each node was measured by calculating bootstrap frequencies with 1,000 bootstrap replicates and 10 random sequence addition replicates. Characters 55, 71, and 91 were treated as ordered (following the original Mayr and Clarke dataset [4]).

Bayesian phylogenetic analyses were run using *MrBayes* [2] on the CIPRES Science Gateway [6], using the *Mk* model of morphological evolution [7] with gamma-distributed rate variation and variable coding. All analyses were performed with a sampling frequency of 1,000, two concurrent runs, and four Metropolis-coupled chains (*T***=**0.1**)** for 10 million generations. Characters 55, 71, and 91 were treated as ordered, and analyses were checked for convergence using standard *MrBayes* diagnostics (e.g., PRSF<0.01, mixing between chains >20%) and *Tracer* (v1.5) [8] (e.g., ESS>200). A 25% relative burn-in was implemented for all summary statistics. Supplementary Table 1 provides the full character/taxon matrix.

Given pervasive incongruities between the morphological phylogenetic topology inferred by Mayr and Clarke [4] and recent phylogenomic analyses of neornithine interrelationships (e.g. [9-13]), we analyzed the morphological matrix under a series of hierarchical topological constraints (‘scaffolds’ *sensu* Lee [14]). The same system of constraints was adhered to under parsimony and Bayesian optimality criteria. First, phylogenetic analyses were performed on the unconstrained morphological dataset (Fig. 2A), and subsequent analyses applied variations of the 50% majority-rule topology from Hackett *et al*. [10] as a topological scaffold (Fig. 2B-D). These constrained analyses fixed the phylogenetic interrelationships of all taxa in the Hackett *et al*. [10] majority-rule topology, except those for which *Foro* was considered a potential fossil total-group representative by Olson [15] (i.e., Opisthocomidae, Cuculidae, and Musophagidae). The first scaffold analysis (Backbone 1) did not fix the phylogenetic position of these three taxa, and subsequent analyses (Backbone 2-4) sequentially fixed the position of Opisthocomidae, Opisthocomidae + Cuculidae, and finally Opisthocomidae + Cuculidae + Musophagidae. Additionally, constrained analyses were performed following the newly published topology of Prum *et al*. [13], applying the same methodology. Constraint trees are provided as separate supplementary files.

Several osteological similarities between *Foro* *panarium* and the Hoatzin (*Opisthocomus hoazin*) were noted in the original description of *F. panarium* [15]. The lineage leading to *O. hoazin* may represent the single longest branch in the neornithine tree of life [13]. Given the historical difficulty of identifying the Hoatzin’s extant sister taxon, and its strikingly autapomorphic skeleton, an effort was made to shorten the phylogenetic branch leading to *O*. *hoazin* by including two stem opisthocomids in the phylogenetic analysis: *Hoazinavis lacustris* from the late Oligocene/early Miocene of Brazil, and *Namibiavis senutae* from the late early Miocene of Namibia [3, 16]. The position of these fossils was left unconstrained in all phylogenetic analyses.

Although variations of the core anatomical dataset used to score *F*. *panarium* in this study have been subjected to numerous phylogenetic analyses under parsimony (e.g. [4, 17-19]), to our knowledge, the present study represents the first time this matrix has been analyzed within a Bayesian phylogenetic framework.

Historical biogeographic analyses

To obtain a time-scaled phylogeny, we used a majority-rule consensus tree identified by *Mesquite* [20] based on a sample of 1,000 trees from the posterior distribution of Jetz *et al*. [21] (<http://www.birdtree.org>). This tree also applied the Hackett *et al*. [10] topology as a higher-order phylogenetic scaffold, allowing for direct comparisons with the constrained analyses described above. The timescale for the higher-order diversification of crown birds from Jetz *et al*. [21] has been criticized as being excessively ancient (supporting a major radiation of extant birds in the Mesozoic [12, 13]). However, we elected to perform our comparative biogeographic analyses on this timescale because a Mesozoic radiation of crown birds is a necessary corollary of the vicariant model advocated by Cracraft [22]. Following Cracraft [22], we restricted our sampling to crown neornithine family-level clades whose present-day biogeographic distributions are either exclusively Gondwanan or exclusively Laurasian.

The biogeographic history of birds in the Northern Hemisphere has been complex throughout the Cenozoic. In particular, the European fossil record indicates that several bird groups restricted to lower-latitudes today, including total group Struthionidae, Bucorvidae, and Opisthocomidae, may have migrated into higher latitude environments during a period of warmer global temperatures around the Miocene climatic optimum ~15 MYA, after having arisen in lower-latitude settings [23-25]. In order to avoid conflating Palaeogene biogeographic patterns with the later overprinting of Northern Hemisphere avifaunas by dispersal from the tropics during the Miocene [25], we elected to restrict our fossil sampling to Palaeogene localities.

In order to evaluate the potential influence of considering the Palaeogene bird fossil record in historical biogeographic analyses, we performed two separate analytical reconstructions of the biogeographic origin of the avian crown clade. The first was a quantitative approximation of the scenario put forth by Cracraft [22], incorporating every extant nonpasserine clade traditionally ranked at the family level that exhibits a biogeographic distribution that is exclusively ‘Gondwanan’ or ‘Laurasian’, and no fossils (Fig. 3A). Although certain clades (e.g. Nyctibiidae) extend into southernmost North America, or even slightly beyond (e.g. Cracidae), the northward extensions of their ranges are almost certainly attributable to contiguous dispersal following uplift of the Panamanian isthmus. Accordingly, these clades were scored as Gondwanan, as they were by Cracraft [22]. Other clades, such as the Podargidae (frogmouth) crown group, are found exclusively at lower latitudes, but extend into southeast Asia (not a vestige of Gondwana). These clades were excluded from the Gondwanan-only coding scheme. It should be noted, however, that well-supported stem group podargids are known from the Palaeogene of both Europe and North America; as such, the Palaeogene distribution of the Podargidae total-group greatly exceeded the comparatively relictual range occupied by the crown clade today (e.g. [17, 26]). The same is true of several other extant pantropical groups, including Trogonidae and Psittacidae (both of which have extensive Northern Hemisphere fossil records [25]).

The second analysis (Fig. 3B) builds upon the first by incorporating all well-supported extinct Palaeogene sisters to the extant clades in Fig. 3A. For a complete list of the fossil taxa included, see Table 1. Although fossil tip-dating [27] has been used profitably to investigate the influence of fossils on ancestral state reconstructions (e.g. [28]), owing to the lack of a sufficiently taxonomically-inclusive anatomical phylogenetic dataset for the avian crown, a ‘total-evidence’ phylogenetic analysis explicitly incorporating both nucleotide sequences and anatomical data for crown birds, and fossil data for Palaeogene representatives, was beyond the scope of this investigation. Instead, we incorporated Palaeogene fossils into the Jetz *et al*. [21] time-calibrated consensus tree by grafting them into their phylogenetic positions as inferred by independent phylogenetic analyses (Table 1); the ages of the fossils grafted into the consensus tree followed the age of the earliest well-supported representative of the fossil species (Table 1). The node subtending each grafted fossil and its extant sister clade was placed 20 million years prior to the age of the fossil, except for the nodes within total-clade Spheniscidae and Anseranatidae, because this would conflict with the age for the node subtending crown Spheniscidae and Anseranatidae and their extant sisters from the Jetz *et al*. [21] analysis. For these clades, a five million-year offset was applied instead.

We tested the robustness of the hypothesis that birds originated in the Southern Hemisphere by performing ancestral area reconstructions in a Bayesian framework using the *R* (v. 2.15.1) package *phytools* (function *make.simmap*) [29], under the equal rates (ER) model with an estimated stationary prior distribution on the root, empirical rate matrix, and 500 simulation replicates. Extant and extinct taxa were coded as either ‘Gondwanan’ or ‘Laurasian’, and the results of these reconstructions are presented at the nodes of Fig. 3A and 3B.

Although numerous model-based methods for historical biogeographic analysis exist, we elected to adhere to a simple analytic paradigm for our biogeographic analyses. Sophisticated methods such as Lagrange [30] require introducing a relative dispersal probability matrix among different areas through time. However, establishing such a matrix may not be ideal for reconstructing biogeographic history across large stretches of geological time, due to a breakdown of the conceptual foundation of these methods when predefined geographical areas change significantly through time (as has been the case with the breakup of the Gondwanan supercontinent [28]). The fact that India, a Gondwanan derivative, is now welded to Laurasia provides a conspicuous case in point. As discussed extensively by Hsiang *et al.* [28], dispersal to and from a continental landmass whose identity and geographic position have changed markedly due to continental breakup and drift causes a breakdown of the conceptual underpinnings of many model-based biogeographic methods, and these problems may be aggravated by organisms with high dispersal capacities, as is likely the case with birds. Because these issues have been demonstrated to be analytically challenging when addressing the ancient biogeographic history of highly dispersive organisms [28], our model-based analyses rely on simple maximum likelihood estimates of ancestral ranges.

Supplementary Fig. 1 presents results of alternative parameterizations of the historical biogeographic analyses. Crown Todidae were coded as Laurasian in the following analyses: 2, 3, 4, 8 (they are endemic to the Greater Antilles). They were coded as Gondwanan in analyses 1, 5, 6, 7 (in case their presence in the Northern Hemisphere reflects a South American origin and subsequent restriction of their distribution to the Greater Antilles). Fossil stem opisthocomids were included in analyses 3-6; they were coded as Gondwanan in analyses 3 and 5, and Laurasian in analyses 4 and 6. Alternative codings reflect the fact that the oldest-known stem opisthocomid is *Protoazin* *parisiensis* from the late Eocene of Europe, but the most stemward total-clade opisthocomid yet known is *Namibiavis senutae*, from the middle Miocene of Namibia. In analysis 7 and 8, both stem Todidae and stem Anseranatidae were removed from the fossil dataset, owing to potential ambiguity surrounding their phylogenetic position. In analysis 9, Todidae was coded as Gondwanan, and all fossils were removed (in contrast to Fig. 3A, where Todidae was coded as Laurasian). In all analyses with fossils, ambiguous biogeographic reconstructions are inferred for the root node.

References

1. Swofford D.L. 2002 PAUP* Phylogenetic Analysis Using Parsimony (*and Other Methods) v.4.0b10 for Macintosh (Sinauer Associates; Sunderland). (

2. Ronquist F., Huelsenbeck J.P. 2003 MRBAYES 3: Bayesian phylogenetic inference under mixed models. *Bioinformatics* **19**, 1572-1574.

3. Mayr G., Alvarenga H., Mourer-Chauviré C. 2011 Out of Africa: Fossils shed light on the origin of the hoatzin, an iconic Neotropic bird. *Naturwissenschaften* **98**(11), 961-966.

4. Mayr G., Clarke J. 2003 The deep divergences of neornithine birds: a phylogenetic analysis of morphological characters. *Cladistics* **19**(6), 527-553.

5. Mayr G., Alvarenga H., Mourer-Chauviré C. 2011 Out of Africa: Fossils shed light on the origin of the hoatzin, an iconic Neotropic bird. *Naturwissenschaften* **98**, 961-966. (doi:DOI 10.1007/s00114-011-0849-1).

6. Miller M.A., Pfeiffer W., Schwartz T. 2010 Proceedings of the Gateway Computing Environments Workshop. (pp. 1-8.

7. Lewis P.O. 2001 A likelihood approach to estimating phylogeny from discrete morphological character data. *Syst Biol* **50**, 913-925.

8. Drummond A.J., Suchard M.A., Xie D., Rambaut A. 2012 Bayesian phylogenetics with BEAUti and the BEAST 1.7. *Molecular biology and evolution* **29**(8), 1969-1973.

9. Ericson P.G.P., Zuccon D., Ohlson J.I., Johansson U.S., Alvarenga H., Prum R.O. 2006 Higher level phylogeny and morphological evolution of tyrant flycatchers, cotingas, manakins and their allies (Aves: Tyrannida). *Mol Phylogenet Evol* **40**, 471 - 483.

10. Hackett S.J., Kimball R.T., Reddy S., Bowie R.C.K., Braun E.L., Braun M.J., Chojnowski J.L., Cox W.A., Han K.-L., Harshman J., et al. 2008 A phylogenomic study of birds reveals their evolutionary history. *Science* **320**, 1763-1768.

11. McCormack J.E., Harvey M.G., Faircloth B.C., Crawford N.G., Glenn T.C., Brumfield R.T. 2013 A phylogeny of birds based on over 1,500 loci collected by target enrichment and high-throughput sequencing. *PLoS One* **ci**. (doi:10.1371/journal.pone.0054848).

12. Jarvis E.D., Mirarab S., Aberer A.J., Li B., Houde P., Li C., Ho S.Y.W., Faircloth B.C., Nabholz B., Howard J.T., et al. 2014 Whole-genome analyses resolve early branches in the tree of life of modern birds. *Science* **346**(6215), 1320-1331. (doi:10.1126/science.1253451).

13. Prum R.O., Berv J.S., Dornburg A., Field D.J., Townsend J.P., Lemmon E.M., Lemmon A.R. 2015 A comprehensive phylogeny of birds (Aves) using targeted next-generation DNA sequencing. *Nature* **526**(7574), 569-573. (doi:10.1038/nature15697

[http://www.nature.com/nature/journal/v526/n7574/abs/nature15697.html - supplementary-information)](http://www.nature.com/nature/journal/v526/n7574/abs/nature15697.html#supplementary-information)).

14. Lee M.S.Y. 2013 Turtle origins: insights from phylogenetic retrofitting and molecular scaffolds. *J Evol Biol* **26**, 2729-2738.

15. Olson S.L. 1992 A new family of primitive landbirds from the Lower Eocene Green River Formation of Wyoming. In *Papers in avian paleontology honoring Pierce Brodkorb* (ed. Campbell K.E.), pp. 137-160, Natural History Museum of Los Angeles County Science Series.

16. Mayr G., De Pietri V.L. 2014 Earliest and first Northern Hemisphere hoatzin fossils substantiate Old World origin of a "Neotropic endemic". *Naturwissenschaften*. (doi:DOI 10.1007/s00114-014-1144-8).

17. Nesbitt S.J., Ksepka D.T., Clarke J.A. 2011 Podargiform Affinities of the Enigmatic *Fluvioviridavis platyrhamphus* and the Early Diversification of Strisores (“Caprimulgiformes” + Apodiformes). *PLoS ONE* **6**(11), e26350. (doi:10.1371/journal.pone.0026350.g008).

18. Bertelli S., Lindow B.E.K., Dyke G.J., Mayr G. 2013 Another charadriiform-like bird from the lower Eocene of Denmark. *Paleontological Journal* **47**(11), 1282-1301. (doi:10.1134/S0031030113110026).

19. Ksepka D.T., Clarke J.A., Nesbitt S.J., Kulp F.B., Grande L. 2013 *Fossil evidence of wing shape in a stem relative of swifts and hummingbirds (Aves, Pan-Apodiformes)*.

20. Maddison W.P., Maddison D.R. 2011 Mesquite: a modular system for evolutionary analysis. Version 2.75. In <http://mesquiteprojectorg> (

21. Jetz W., Thomas G.H., Joy J.B., Hartmann K., Mooers A.O. 2012 The global diversity of birds in space and time. *Nature* **491**, 444-448. (doi:doi:10.1038/nature11631).

22. Cracraft J. 2001 Avian evolution, Gondwana biogeography and the Cretaceous-Tertiary mass extinction event. *Proceedings of the Royal Society B: Biological Sciences* **268**(1466), 459-469. (doi:10.1098/rspb.2000.1368).

23. Mourer-Chauviré C. 1999 Les relations entre les avifaunes du Tertiaire inférieur d'Europe et d'Amérique du Sud. *Bulletin de la Société Géologique de France* **170**(1), 85-90.

24. Brunet J. 1970 Oiseaux de l’Éocène supérieur du bassin de Paris. *Ann Paleontol* **56**, 3-57.

25. Mayr G. 2011 Two-phase extinction of "Southern Hemispheric" birds in the Cenozoic of Europe and the origin of the Neotropic avifauna. *Palaeobiodiversity and Palaeoenvironments* **91**, 325-333. (doi:10.1007/s12549-011-0062-4).

26. Mayr G. 2015 Eocene fossils and the early evolution of frogmouths (Podargiformes): further specimens of Masillapodargus and a comparison with Fluvioviridavis. *Palaeobiodiversity and Palaeoenvironments*, 1-10. (doi:10.1007/s12549-015-0200-5).

27. Ronquist F., Klopfstein S., Vilhelmsen L., Schulmeister S., Murray D.L., Rasnitsyn A.P. 2012 A Total-Evidence Approach to Dating with Fossils, Applied to the Early Radiation of the Hymenoptera. *Systematic Biology*. (doi:10.1093/sysbio/sys058).

28. Hsiang A.Y., Field D.J., Webster T.H., Behlke A.D.B., Davis M.B., Racicot R.A., Gauthier J.A. 2015 The origin of snakes: revealing the ecology, behavior, and evolutionary history of early snakes using genomics, phenomics, and the fossil record. *BMC Evolutionary Biology* **15**(1), 1-22. (doi:10.1186/s12862-015-0358-5).

29. Revell L.J. 2012 Phytools: An R package for phylogenetic comparative biology (and other things). *Methods Ecol Evol* **3**, 217-223.

30. Ree R.H., Moore B.R., Webb C.O., Donoghue M.J. 2005 A likelihood framework for inferring the evolution of geographic range on phylogenetic trees. *Evolution* **59**(11), 299–311.
