# Supplementary material for: A North American stem turaco, and the complex biogeographic history of modern birds
Source: BMC Evol Biol. 2018 Jun 25;18:102. doi: 10.1186/s12862-018-1212-3 (PMC6016133; doi:10.1186/s12862-018-1212-3)
Supplement: Supplementary file 3 — Table S1. Character/taxon matrix used for the phylogenetic analyses. Character descriptions follow Mayr 2011 for characters 6, 15, 22, 31, 32, 44, 57, 58, 65, 67, 85, 94, 100, 105, 106, 149, 150, and 151. For the remaining characters, descriptions follow Mayr & Clarke 2003, except newly added characters 152 and 153 as described in the main text. (PDF 123 kb) [file 12862_2018_1212_MOESM3_ESM.pdf]

Taxon	1	2	3	4	5	6	7	8
Rheidae	1	0	0	1	0	0	0	0
Apterygidae	1	0	0	1	0	0	0	1
Tinamidae	1	0	0	1	0	0	0	0
Galliformes	1	0	0	0	0	0	0	0
Anhimidae	1	0	1	0	0	0	0	0
Anatidae	1	0	2	0	0	0	0	0
Podicipedidae	1	0	0	0	0	0	0	0
Phoenicopteridae	1	0	2	1	0	0	0	0
Threskiornithidae	1	0	0	1	0	1	0	0
Cariamidae	1	0	0	0	0	0	0	0
Strigiformes	1	1	0	0	1	0	0	1
Recurvirostridae	1	0	0	0	0	1	0	0
Burhinidae	1	0	0	0	0	0	0	0
Accipitridae	1	1	0	0	0	0	0	1
Falconidae	1	1	0	0	0	0	0	1
Sagittariidae	1	1	0	0	0	0	0	1
Cathartidae	1	1	0	0	0	0	0	0
Gaviidae	1	0	0	0	0	0	0	0
Spheniscidae	1	0	0	1	0	0	0	0
Phaethontidae	1	0	0	0	1	0	0	0
Fregatidae	1	1	0	1	0	0	1	?
Phalacrocoracidae	1	1	0	1	1	0	1	?
Balaenicipitidae	1	1	0	1	1	0	1	?
Eurypygidae	1	0	0	0	0	1	0	0
Ardeidae	1	0	0	0	0	0	0	0
Ciconiidae	1	0	0	0	0	0	0	0
Procellariidae	1	1	0	1	0	0	0	0
Otididae	1	0	0	0	0	0	0	0
Pteroclidae	1	0	0	0	0	0	0	0
Columbidae	1	0	0	0	0	1	0	0
Rallidae	1	0	0	0	0	0	0	0
Psophiidae	1	0	0	0	0	0	0	0
Gruidae	1	0	0	0	0	1	0	0
Steatornithidae	1	1	0	0	1	0	0	1
Psittacidae	1	1	0	0	1	0	0	1
Coliidae	1	0	0	0	0	0	0	1
Trogonidae	1	0	0	0	1	0	0	1
Aegothelidae	1	1	0	0	0	0	0	0
Coraciidae	1	0	0	0	0	0	0	1
Passeriformes	1	0	0	0	0	0	0	0
Namibiavis	?	?	?	?	?	?	?	?
Hoatzinavis	?	?	?	?	?	?	?	?
Musophagidae	1	0	0	0	0	0	0	1
Cuculidae	1	0	0	0	0	0	0	1
Opisthocomidae	1	0	0	0	1	0	0	0
Foro panarium	1	0	?	0	?	0	0	0

9	10	11	12	13	14	15	16	17
0	1	0	0	1	0	0	0	0
0	1	0	0	0	0	0	0	0
0	1	0	0	0	0	0	0	0
0	0	0	1	0	0	0	0	0
0	0	1	1	0	?	0	1	1
0	0	1	1	0	?	0	0	1
0	0	0	1	0	0	1	1	0
0	0	1	0	0	?	1	1	0
0	0	1	1	0	0	1	1	0
0	0	1	1	1	0	1	1	0
0	0	0	0	1	0	1	1	0
0	0	0	1	0	0	1	1	0
0	0	0	0	0	?	1	1	0
0	0	0	0	0&1	0	1	1	0
0	0	0	0	1	0	1	1	0
0	0	0&1	0	1	0	0	1	0
0	0	0	0	0	0	1	1	0
0	0	0	0	0	?	1	1	0
0	0	0	0	0	0	0	1	0
0	0	0	0	0	0	1	0	0
1	0	0	0	0	?	1	1	1
1	0	1	0	0	?	1	1	0&1
0	0	1	0	0	?	1	1	1
0	0	0	1	0	?	1	1	0
0	0	0&1	0	0	0	1	1	0
0	0	1	1	0	?	1	1	0
0	0	0	0	0	0	1	1	0
0	0	0	0	0	0	1	1	0
0	0	0	0	0	1	0	0	0
0	0	0	0	0	1	0	1	0
0	0	0	0	1	0	1	1	0
0	0	0	0	0	0	0	1	0
0	0	0	0&1	1	0	1	1	0
0	0	1	1	0	0	0	0	1
0	0	1	0	0&1	0	0	1	0
0	0	1	1	0	1	0	1	0
0	0	0	0	0	0	0	0	1
0	0	0	?	?	0	0	1	0
0	0	1	0	0	0	1	1	1
0	0	0	0	0	0	0	0	0
?	?	?	?	?	?	?	?	?
?	?	?	?	?	?	?	?	?
0	0	0	0	0	0	0	0	0
0	0	1	0	0	1	1	1	0
0	0	0	0	0	0	0	0	0
?	?	?	?	?	0	?	?	?

18	19	20	21	22	23	24	25	26
0	0	0	0	0	0	0	0	0
0	0	0	0	0	0	0	0	0
0	0	0	0	0	0	0	0	0
0	1	1	1	1	0	1	0	1
0	1	1	1	1	0	1	0	1
0	1	1	1	1	0	1	0	1
0	0	1	1	1	1	0	0	0
0	1	0	1	1	1	0	0	0
0	1	1	0	1	1	0	0	0
0	1	1	1	1	1	0	0	0
1	?	?	?	1	0	0	0	0
0	0	0	0	1	0	0	1	0
0	0	1	0	1	1	0	0	0
0	1	1	1	1	1	0	0	0
0	0	1	0	1	1	0	0	0
1	?	?	?	1	0	0	0	0
1	?	?	?	1	0	0	0	0
0	0	1	1	1	1	0	1	0
0	0	1	1	1	1	0	1	0
0	1	0	0	1	1	0	0	0
0	1	1	0	1	1	0	0	0
1	?	?	?	1	1	0	0	0
1	?	?	?	1	1	0	0	0
0	0	1	0	1	1	0	0	0
0	0	1	1	1	1	0	0	0
1	?	?	?	1	1	0	0	0
0	0	0	0	1	0	0	1	0
0	1	1	1	1	1	0	0	0
1	?	?	?	1	0	0	0	0
1	?	?	?	1	0	0	0	0
0	0	1	1	1	1	0	0	0
0	0	1	1	1	1	0	0	0
0	0	0	1	1	1	0	0	0
1	?	?	?	1	0	0	0	0
1	?	?	?	1	1	0	0	0
1	?	?	?	1	1	0	0	0
0	1	1	0	1	0	0	0	0
0	0	0	0	1	0	0	0	0
0	1	1	0	1	1	0	0	0
0	0	0	0	1	1	0	0	0
?	?	?	?	?	?	?	?	?
?	?	?	?	?	?	?	?	?
0	0	1	0	1	1	0	0	0
1	?	?	?	1	1	0	0	0
0	1	1	0	1	1	0	0	0
?	?	?	?	?	?	?	0	?

27	28	29	30	31	32	33	34	35
0	0	0	0	1	1	0	0	0
0	0	0	1	1	1	0	0	0
0	0	0	0	1	0	0	0	0
0	0	1	0	0	1	0	0	1
0	0	1	0	0	1	1	1	1
1	0	1	0	0	1	1	1	1
0	0	1	1	0	1	0	1	0
1	0	1	1	1	1	0	1	0
1	0	1	1	0	1	0	1	0
0	0	1	0	1	1	0	1	0
0	0	1	0	0	1	0	1	0
1	0	1	0	0	1	0	1	0
0	0	1	0	0	1	0	1	0
0	0	1	0	0	1	0	1	0
0	0	1	0	0	1	0	1	0
0	0	1	0	0	1	1	1	0
0	1	1	1	0	1	0	1	0
0	0	1	1	0	1	0	1	0
0	0	1	1	0	1	1	1	1
0	0	1	1	0	1	1	1	0
0	1	1	1	0	1	1	1	0
0	0	1	1	0	1	1	1	0
0	1	1	0	1	1	0	1	0
0	0	1	0	1	1	1	1	0
0	0	1	0	0	1	1	1	0&1
0	0&1	1	1	0	1	0	1	0
0	1	1	1	0	1	1	1	0
0	0	1	0	1	1	0	1	0
0	0	1	0	0	1	0	1	0
0	0	1	0	1	1	1	1	0
0	0	1	0	1	1	0	1	0&1
0	0	1	0	0	1	0	1	0
1	0	1	0	1	1	1	1	0
0	0	1	0	0	1	1	1	0
0	0	1	0	0	1	1	1	0
0	0	1	0	1	1	1	1	0
0	0	1	0	0	1	1	1	0
0	0	1	0	0	1	1	1	0
0	0	1	0	0	1	0	1	0
0	0	1	0	0&1	1	0	1	0
?	?	?	?	?	?	?	?	?
?	?	?	?	?	?	?	?	?
0	0	1	0	1	1	1	1	0
0	0	1	0	0&1	1	0	1	0
0	0	1	0	1	1	0	1	0
?	0	1	?	1	?	?	?	0

36	37	38	39	40	41	42	43	44
1	0	0	?	1	1	1	1	0
0	0	0	0	1	1	1	1	0
1	0	0	0	1	1	1	1	0
0	0	1	0	0	1	1	0	2
0	0	1	0	0	1	1	0	1
0	0	1	0	0	1	1	0	1
0	1	0	0	0	1	1	0	0
0	1	0	0	0	1	1	0	1
0	1	0	0	0	1	1	0	2
1	0	0	0	0	1	1	0	0
0	0	0	0	0	1	1	0	0
0	1	0	0	0	1	1	0	2
1	1	0	0	0	1	1	0	0
0	0	0	0	0	1	1	0	0
0	0&1	0	0	0	1	1	0	0
1	0	0	0	0	1	1	0	0
0	1	0	0	0	1	1	0	0
0	1	0	0	0	1	1	0	0
0	1	0	0	0	1	1	0	0
0	0	0	0	0	1	1	0	0
0	0	0	0	0	1	1	0	0
0	1	0	0	0	1	1	0	0
0	1	0	0	0	1	1	0	0
0	1	0	0	0	1	1	0	0
0	1	0	0	0	1	1	0	0
1	1	0	0	0	1	1	0	0
0&1	1	0	0	0	1	1	0	0
0	0	0	0	0	1	1	0	0
0	0	0	0	0	1	1	0	1
0	0	0	0	0	1	1	0	0
0	1	0	0	0	1	1	0	2
1	1	0	0	0	1	1	0	0
1	1	0	0	0	1	1	0	2
0	0	0	1	0	1	1	0	0
0	0	0	0	0	1	1	0	0
1	0	0	0	0	1	1	0	0
0	0	0	1	0	1	1	0	0
1	0	0	0	0	1	1	0	0
0	0	0	0	0	1	1	0	0
0	0	0	0	0	1	1	0	0
?	?	?	?	?	?	?	?	?
?	?	?	?	?	?	?	?	?
0	0	0	0	0	1	1	0	0
0&1	0	0	0	0	1	1	0	0
1	0	0	0	0	1	1	0	0
?	?	?	?	0	1	1	0	0

45	46	47	48	49	50	51	52	53
0	0	0	0	0	0	0	0	0
0	0	0	1	1	0	0	1	0
0	0	0	1	0	0	0&1	0	0
1	0	0	0	0&1	1	0	1	0
1	0	0&1	1	0	0	0&1	1	0
1	0	0&1	0&1	0	0	0	1	0
0	1	0	0	0	0&1	0	0	0
0	1	0	1	1	1	0	0	0
0	0&1	0	1	1	0	0	1	0
0	0	0	1	0	1	0	1	0
1	0	0	0	1	1	0	1	0
0	0	0	0	1	1	0	1	0
0	0	0	0	0	0	0	1	1
1	0	0	0	1	0	0	0&1	0
1	0	0	0	1	1	0	1	0
1	1	1	0	0	1	1	1	0
1	0	0	0	0&1	1	0	1	0
0	1	0	0	1	1	0	0	0
0	0	0	0	1	1	0	0	0
0	1	0	1	1	1	0	1	0
0	1	0	0	1	1	0	1	0
1	1	0	0	1	0	0	0	0
0	0	0	1	0	1	0	0	0
0	0	0	1	0	1	0	0	0
0	0	0	1	0	1	0	0	0
0	0	0&1	1	0	0	0	1	0
0	1	0	0	1	1	0	0	0
0	0	0	1	0	1	0&1	0&1	0
1	0	0	0	1	1	0	1	0
0	0	0	0	1	1	0	0	0
0	0	0	0	1	1	0	0&1	0
0	0	0	0	0	1	0	0	0
0	0	0	1	0	0	0	0	0
0	0	0	0	0	1	0	0	0
0	1	0	0	1	1	0	0&1	0&1
0	1	0	0	1	1	0	1	1
1	0	0	0	1	1	0	0	0
0	1	0	0	1	1	0	0	0
0	0	0	0&1	1	0&1	1	1	1
1	0	0	0	1	1	0	1	0
?	?	?	?	?	?	?	?	?
?	?	?	?	?	?	?	?	?
0	0	0	0	0&1	1	0	1	1
1	0	0	0	0&1	0	0	1	0&1
0	0	1	1	0	1	1	1	0
?	?	0	?	?	1	0	1	?

54	55	56	57	58	59	60	61	62
0	1	0	1	0	0	0	0	?
0	1	0	1	0	0	0	0	?
0	1	1	1	0	0	0	0	0
0	1	1	1	0&1	0	0	1	0
0	1	0	1	0	1	0	1	0
0	1	0	1	0	1	0	1	0
1	2	1	1	0	1	0	1	0
1	2	1	1	0	1	0	0	0
0	1	1	1	0	1	0	0	0
0	0	0	1	0	1	0	0	0
0	0	0	1	0	1	0	0	0&1
0	0	0	0	1	1	0	1	0
0	0	0	0	1	1	0	1	0
0	0&1	0	1	0	1	0	0	1
0	0&1	1	1	0	1	0	0	1
0	0	0	1	0	1	0	0	0
0	1	0	1	0	1	0	0	0
0	1	0	1	0	1	0	1	0
0	1	0	0	0	1	0	0	0
0	2	0	1	0	1	?	?	0
0	0	0	1	0	1	1	0	?
0	2	0	0	0	1	1	0	1
0	1	0	1	0	1	0	1	1
0	2	1	1	0	1	0	1	0
0	2	0	1	0	1	0	1	0
0	1	0	1	0	1	0	0	0
0	1	0	1	1	1	0	0	0
0	1	0	1	0	0	0	1	0
0	1	1	1	0	1	0	1	0
0	0	1	1	0	1	0	1	0
0	1&2	0	1	0	1	0	1	0
0	2	1	1	0	0	0	1	0
0	2	1	1	0	0	0	1	0
0	0	1	0	0	1	1	1	0
0	0	0	1	0&1	1	0	1	0
0	0	0	1	0	1	1	0	0
0	0	0	1	0	1	1	1	0
0	0	0	1	0	1	1	1	0
0	0	0	1	0	1	1	1	0
0	0	0	1	0	1	1	1	0
?	?	?	?	?	?	?	?	?
?	?	?	?	?	?	?	?	?
0	0	0	1	0	1	1	0	1
0	0	0	1	0	1	0	1	0
0	1	1	1	0	1	0	0	1
0	0	0	?	?	?	?	1	0

63	64	65	66	67	68	69	70	71
?	0	1	0	0	1	0	0	0
?	0	0	0	0	1	0	0	0
0	0	1	1	0	0	0	0	0
0	0	1	0	0	0	0	1	0
0	0	0	0	2	0	0	0	2
0	0	1	0	0	0	0	0&1	1&2
0	0	1	0	0	0	0	0	1
0	0	0	0	0	0	1	1	1
0	0	0	0	0	0	1	1	1
0	0	1	0	0	0	0	1	1
0	0	0	0	0	0	0&1	0	1
0	1	0	0	0	0	0	1	1
0	1	0	0	0	0	0	1	1
0	0	0	0	0	0	0&1	0&1	1
0	0	0	0	0	0	1	1	1
1	0	0	0	0	0	0	0	1
0	0	0	0	1	0	0	0	1
0	0	0	0	0	0	0	0	2
0	0	0	0	0	0	0	1	1
1	0	0	0	0	0	1	1	1
1	0	1	0	0	0	0	0	1
1	0	1	0	0	0	1	0	1
1	0	0	0	0	0	1	0	1
0	0	1	0	0	0	1	1	1
0	0	1	0	0	0	1	1	0
1	0	0&1	0	0	0	0&1	0	1
0	0	0	0	0	0	1	1	1
0	0	1	1	0	0	0	0	1
0	0	1	0	0	0	0	0	1
0	0	1	0	0	0	0	0&1	0
0	0	0	0	0&1	0	0	1	1
0	0	0	0	1	0	0	0	2
0	0	0	0	2	0	0&1	0	2
0	0	1	0	0	0	0	0	0
0	0	0&1	0	0	0	1	1	1
0	0	0&1	0	0	0	0	1	0
0	0	1	0	0	0	0	1	0
0	0	0	0	0	0	0	0	0
0	0	1	0	0	0	0	1	1
0	0	1	0	0	0	0	1	1
?	0	1	0	2	0	?	?	?
?	0	1	0	?	0	?	?	?
0	0	0	0	0	0	1	1	0&1
0	0	1	0&1	0	0	0	1	0
0	0	1	1	2	0	?	?	1
0	0	?	?	?	0	?	1	1

72	73	74	75	76	77	78	79	80
0	2	0	0	?	0	0	?	?
0	1	0	0	?	0	0	?	?
0	1	1	0	1	1	0	0	0
0	0	1	0	1	1	0	1	0
0	1	2	0	0	1	0	1	0
0	1	1	0	0	1	0	1	0
0	1	0	0	0	0	1	1	0
1	1	1	0	0	1	1	1	0
0	0	1	0	0	1	0	1	0
0	1	1	0	0	1	0	1	0
0	0	1	0	0	1	0	1	0
0	0	1	0	0	0	0	1	0
0	0	1	0	0	0	0	1	0
0	1	1	0	0	1	0	1	0
0	1	1	0	0	1	0	1	0
1	2	1	0	0	1	0	1	1
0	0	1	0	0	1	0	1	1
0	1	0	0	0	0	0	1	0
0	1	0	0	0	0	0	?	?
0	2	1	0	0	1	0	1	0
1	2	1	0	0	1	0	1	0
0	1	1	0	0	0	0	1	0
1	1	1	0	0	1	0	1	0
0	1	1	0	0	1	0	1	0
0	1	1	0	0	1	0	1	0
1	1	1	0	0	1	0	1	0
0&1	0	1	0	0	0	0	1	1
0	0	1	0	0	1	0	1	0
0	0	1	1	1	1	0	1	0
0	0&1	1	1	1	1	0	1	0
0	1	1	0	0	0	0	1	0
1	2	1	0	0	1	0	1	0
1	2	1	0	0	1	0	1	0
0	1	1	0	0	1	0	1	1
0	1	1	0	0	1	0	1	0
0	0	1	0	0	1	0	1	0
0	0	1	0	0	1	0	1	1
0	0	1	0	0	1	0	1	0
0	0	1	0	0	1	0	1	0
0	1	1	0	0	1	0	1	0
?	?	?	0	0	?	0	1	0
?	?	?	0	0	1	0	1	1
0	0	1	0	0	1	0	1	0
0	0&1	1	0	0	1	0	1	0
1	1	1	0	0	1	0	1	1
?	0	1	0	0	1	0	1	0

81	82	83	84	85	86	87	88	89
?	0	?	?	0	0	?	?	?
?	0	?	?	0	0	?	?	?
0	0	0	0	0	0	0	0	0
1	0	0	0	0	0	0	0	0
1	0	1	1	0	0	0	0	0
1	0	1	0&1	0	0	0	0	0
1	0	1	1	0	0	0	0	1
1	0	1	1	0	0	0	0	1
1	1	1	0	0	0	0	0	0
1	0	0	0	1	1	0	0	0
1	1	1	0	0	0	0	0	0
1	1	1	0	0	0	0	1	0
1	1	1	0	0	0	0	1	0
1	1	1	1	0	0	0	0	0
1	1	1	1	0	0	0	0	0
1	0	1	0	0	0	0	0	0
1	1	1	0	0	0	0	0	0
1	0	1	0	0	0	0	0	0
?	0	?	?	0	0	0	0	0
1	1	1	0	0	0	0	0	0
1	1	1	0	0	0	0	0	1
1	1	1	0	0	0	0	0	1
1	1	1	0	0	0	0	0	0
1	1	1	0	0	0	0	0	0
1	1	1	0	0	0	0	0	0
1	1	1	0&1	0	0	0	0&1	0
0	0	1	0	0	0	0	0	1
1	0	1	0	0	0	0	0	0
1	1	1	0	1	0	0	0	0
1	1	1	0	1	0	0	1	0
1	0	0	0	0	0	0	0	0
1	0	0	0	1	0	0	0	0
1	1	1	0	0	0	0	0	0
1	1	1	1	0	0	0	0	0
1	1	1	1	0	0	0	0	0
1	0&1	1	0	1	0	0	0	0
1	1	1	0	1	0	0	0	0
1	1	1	0	0	0	0	0	0
1	1	1	0	0	0	0	0	0
1	1	1	0	0	0	0&1	0	0
1	?	?	?	?	?	?	?	?
-	?	?	?	?	?	?	?	?
1	0	1	0	1	1	1	0	0
1	0	1	0	0&1	0&1	1	0	0
1	0	1	0	1	1	0	0	0
1	0	1	?	1	1	1	0	0

90	91	92	93	94	95	96	97	98
0	?	1	0	1	0	0	0	0
0	2&3	1	0	0	0	0	0	0
0	4	0	0	0	0	0	1	0
0	3	1	0	1	1	0	1	0&1
0	4	1	1	1	0	1	0	1
0	3&4	1	0	1	0	0&1	0	0
1	4	0	1	1	0	1	0	0
0	2	1	1	1	0	1	1	1
0	3	1	1	1	0	0	1	0
0	2	1	1	1	1	0	1	0
0	2	1	1	1	1	0	0	0
0	2	0	1	1	0	0	1	0
0	3	0	1	1	0	0	1	0
0	2	1	1	1	0	0	0	1
0	1&2	1	1	1	0	0	1	1
0	2	1	1	1	1	0	1	1
0	2	1	1	1	0	0	1	1
1	4	0	1	1	0	1	0	0
0	1	0	1	1	0	0	0	0
0	1	0	1	1	0	0	0	0
0	2	0	1	1	0	1	0	0
0	3	1	1	1	0	0	0	0
0	3	1	1	1	?	0	0	0
0	2	0	1	1	0	0	0	0
0	2	1	1	1	0	0	0	0
0	3	1	1	1	0	1	1	1
0	2	0	1	1	0	0	0	0
0	3	0	1	1	?	1	1	1
0	2	0	1	1	0	0	1	0
0	2	0	1	1	0	0	1	0
0	4	1	1	1	1	0	1	0
0	3	1	1	1	1	0	1	0
0	3	1	1	1	1	0	1	0
0	1	0	1	1	0	0	0	0
0	2	1	1	1	0	0	0	0
0	1	0	1	1	0	0	0	0
0	1	0	1	1	0	0	0	0
0	0	0	1	1	0	0	0	0
0	1	0	1	1	0	0	0	0
0	1	0	1	1	0	0	0	0
?	?	?	?	?	?	?	?	?
?	?	?	?	?	?	?	?	?
0	2	0	0	1	1	0	0	1
0	1	0&1	0&1	1	0&1	0	0	0
0	3	1	1	1	1	0	0	0
0	?	?	0	?	?	0	0	?

99	100	101	102	103	104	105	106	107
0	0	0	0	0	0	0	0	0
0	0	0	0	0	0	0	0	1
0	1	0	0	0	0	0	0	0
0	1	0	0	1	0	1	0	0
0	1	0	0	1	0	0	0	0
0	1	0	0	1	0	0	0	0
1	0&1	0	1	1	1	1	?	0
1	1	1	1	1	1	0	0	0
0	1	0	1	1	1	0	0	0
0	1	0	0	0	0	0	0	0
0	0	0	0	1	0	0	0	0
1	1	0	1	1	0	0	0	0
0	1	0	1	1	0	0	0	0
0	1	0	0	1	0	0	0	0
0	1	0	0	1	0	0	0	0
0	1	0	0	0	0	0	0	0
0	1	0	0	0	0	0	0	0
1	1	0	0	1	1	1	?	0
1	1	0	0	0	0	0	0	1
0	1	0	0	1	1	0	0	0
0	1	0	0	1	0	1	0	0
1	1	0	0	1	0	1	1	0
0	1	0	0	1	0	1	1	0
0	1	0	0	1	0	0	0	0
0	1	0	0	1	0	1	0&1	0
0	1	1	0&1	1	1	0	0	0
1	1	0	1	1	0	0	0	0
0	1	0	0	1	0	1	0	0
0	1	0	0	1	0	1	0	0
0	1	0	0	1	0	1	0	1
0	1	0&1	0	1	0	0&1	0	0
0	1	1	1	1	0	0	0	0
0	1	1	1	1	0	1	0	0
0	0	0	0	1	0	0	0	0
0	0&1	0	0	1	0	0	0	1
1	1	0	0	1	0	0	1	0
0	1	0	0	1	0	1	1	1
0	1	0	0	1	0	1	0	1
0	1	0	0	1	0	1	0	1
0	1	0	0	1	0	1	1	1
?	?	?	?	?	?	?	?	?
?	?	?	?	?	?	?	?	?
0	1	0	0	1	0	1	0	0
0	1	0	0	1	0	1	1	0
0	0	0	0	1	0	1	0	0
0	1	?	?	1	0	?	0	?

108	109	110	111	112	113	114	115	116
1	0	1	0	0	0	0	?	?
1	0	1	0	0	0	0	0	0
0	0	1	0	0	0	0	0	0
0	0	0	0	0	0&1	0	0	0
1	0	0	0	0	0	0	?	?
0	0	1	1	0	0	0	0	0
0	0	1	0	0	0	0	1	1
0	0	1	1	0	0	0	1	0
1	0	0	0	0	0	?	0	0
1	0	1	0	0	0	0	?	?
1	0	0	0	0	1	0	0	0
0	0	1	0&1	0	0	0	?	?
1	0	1	0	0	0	0	?	?
1	0	0	0	0	1	0	0	0
1	0	0	0	0	1	0	0	0
1	0	0	0	0	0	0	0	0
1	0	0	0	0	0	0	0	0
0	0	1	1	0	0	0	0	0
1	0	1	1	?	0	0	0	0
1	0	0	1	1	0	0	?	?
1	0	0	0	1	0	1	0	0
1	0	0	1	1	0	1	0	0
1	0	0	0	0	0	0	0	0
1	0	0	0	0	0	0	?	?
1	0	0	0	0	0	1	0	0
1	0	0	0	0	0	0	1	0
0	0	1	1	?	0	0	0	0
1	0	1	0	0	0	0	?	?
0	0	1	0	0	0	0	?	?
1	0	0	0	0	0	0	0	1
0	0	0	0	0	0	0	0	0
1	0	0	0	0	0	0	?	?
0	0	1	0	0	0	0	0	0
1	0	0	0	0	0	0	0	1
1	1	0	0	0	0&1	0	1	0
1	0	0	0	0	0	0	1	0
1	0	0	0	0	0	0	1	1
1	0	0	0	0	0	0	0	1
1	0	0	0	0	0	0	0&1	1
1	0	0	0	0	0&1	0	0&1	1
?	?	?	?	?	?	?	?	?
?	?	?	?	?	?	?	?	?
1	0	0	0	0	0	0	?	0
1	1	0	0	0	0	0	0	1
1	0	0	0	0	0	0	0	0
1	0	0	?	?	0	0	?	?

117	118	119	120	121	122	123	124	125
?	0	0	1	0	?	?	?	0
0	0	0	0	0	0	0	0	0
1	0	0	0	0	0	0	0	0
0	0	0	0	0	0	0	0	0
?	0	0	?	?	?	?	0	0
1	1	0	0	0	1	0	0	0
1	1	0	1	0	0	0	0	1
0	0	0	1	0	0	0	0	0
0	0	0	1	0	0	0	0	0
?	?	?	1	?	?	?	?	0
0	1	1	0	1	0	0	0	1
?	?	?	?	0	?	?	?	0
?	?	?	1	?	?	?	?	0
1	1	1	0	1	1	0	0	0
1	1	1	0	1	1	0	0	0
0	0	0	1	0	1	0	0	0
1	0	0	0&1	1	1	0	0	0
1	1	0	0	0	0	0	0	0
0&1	1	0	0	0	0	0	0	0
?	0	0	?	1	?	?	?	?
1	1	1	1	1	0	0	?	0
1	1	0	0	1	?	0	0	0
0	0	0	1	1	0	0	0	1
?	?	?	?	?	?	?	?	?
0	0	0	1	1	1	0	0	1
0	0	0	1	1	0	0	0	0
0	1	1	0	0	0	0	0	0
?	0	0	1	?	?	?	?	0
?	?	?	?	0	?	?	?	0
0	0	0	0	0	0	0	0	0
0	0	0	0	0	1	0	0	0
?	?	?	1	?	?	?	?	0
0	0	0	0	0	?	0	0	0
0	0	0	1	1	?	0	0	1
0	0	0	0	1	1	0	0	0
0	0	0	0	1	1	0	0	1
?	1	?	?	?	?	?	0	1
0	?	?	0	1	?	0	0	1
0	0	0	0	1	0	0	0	1
0	0	0	0	1	1	0	0	1
?	?	?	?	?	?	?	?	?
?	?	?	?	?	?	?	?	?
?	0	0	?	0	?	1	1	0
1	0	0	0&1	1	0	1	1	0
0	0	0	0	0	0	0	1	0
?	?	?	?	?	?	?	?	?

126	127	128	129	130	131	132	133	134
?	?	?	?	?	?	1	?	1
0	0	0	0	0	1	0	0&1	1
0	0	0	0	0	0	0	0	0
0	0	0	0	0	0	1	0	0
?	?	0	0	?	?	?	?	?
1&2	0&1	?	0	0	0&1	0	0	0&1
?	1	0	0	1	1	0	0	1
0	0	0	1	0	0	0	0	1
0	0	0	1	0	0	0	0	0
?	?	?	?	?	?	?	?	?
?	0	0	0	0	1	0	1	0
?	?	?	?	?	?	?	?	?
?	?	?	?	?	?	?	?	?
0	0	0	0	0	1	0	1	0
0	0	0	0	0	1	0	0	0
0	0	0	0	0	0	0	1	0
0	0	0	0	0	0	1	0	0
1	1	0	0	1	0	1	0	1
1	0	0	0	0	1	1	0	1
?	?	0	?	?	?	?	?	?
0	0	0	0	0	1	0	0	0
0	0	0	0	0	?	0	0	0
0	0	0	0	0	0	1	0	0
?	?	0	?	?	?	?	?	?
?	0	0	0	0	1	1	0	0
0	0	0	1	0	0	0	0	0
1	0	0	0	0	1	0	0	0
?	?	?	?	?	?	?	?	?
?	?	?	0	?	0	?	1	?
0	0	0	0	0	0	0	0	0
2	0	0	0	0	0	0	0	0
?	?	?	?	?	?	?	?	?
0	0	0	0	0	0	0	0	0
?	0	?	0	?	0	0	0	0
?	0	1	0	1	1	1	1	0
?	0	1	0	1	1	0	1	0
?	?	0	?	0	?	?	0	0
?	0	?	0	?	1	0	0	0
?	0	0	0	1	1	0	0	0
?	?	0	0	0	1	0	0	0
?	?	?	?	?	?	?	?	?
?	?	?	?	?	?	?	?	?
1	?	?	?	?	0	?	?	?
1	0	0	0	0	1	1	0	0
0	0	0	0	0	1	1	0	0
?	?	?	?	?	?	?	?	?

135	136	137	138	139	140	141	142	143
4	?	?	?	?	?	?	0	0
2	0	1	0	0	?	?	0	0
1	0	0	0&1	0	1	0	0	0
1	0	0	0	0	1	0	0	0
?	?	?	?	?	0	0	0	0
2	0	0	0	0	0	0	0	0
4	0	0	1	?	0	1	0	0
4	0	0	1	0	0	1	0	0
1	0	0	0	0	0	0	0	0
?	?	?	?	?	1	0	0	0
1	1	0	0	0	0	0	0	1
?	?	?	?	?	0	0	0	0
?	?	?	?	?	0	0	0	0
3	1	0	0	0	0	0	0	1
3	1	0	0	0	0	0	0	1
3	1	0	0	0	0	0	0	1
5	1	0	0	0	0	0	0	1
4	0	0	1	?	0	0	0	0
2	0	0	1	0	?	?	0	0
?	?	?	?	?	0	0	0	0
5	0	0	0	0	0	0	1	0
2	1	0	0	0	0	0	1	0
1	0	0	0	0	0	0	0	0
?	?	?	?	?	0	0	0	0
1	0	0	0	0	0	0	0	0
1	0	0	0	0	0	1	0	0
4	0	0	1	?	0	0	0	0
?	?	?	?	?	0	0	0	0
?	?	?	?	?	0	0	0	0
1	0	0	0	1	0&1	0	0	1
1	0	0	0	0	0	0	0	0
?	?	?	?	?	1	0	0	0
1	0	0	0	0	0	0	0	0
?	0	1	0	0	0	0	0	0
9	0	1	0	1	0	0	0	1
5	1	1	0	1	1	0	0	0
8	?	1	?	?	1	0	0	0
?	0	1	0	0	0	0	0	0
5	1	0	0	?	0	0	0	0
7	1&2	1	0&1	0	1	0	0	0
?	?	?	?	?	?	?	?	?
?	?	?	?	?	?	?	?	?
?	?	?	?	?	1	0	0	0
1	0	0	0	0	1	0	0	0
1	0	0	0	0	1	0	0	0
?	?	?	?	?	?	?	?	?

144	145	146	147	148	149	150	151	152
1	0	0	0	0	0	0	0	0
1	?	0	0	0	0	0	0	0
0	0	0	0	0	0&1	0	0	0
0	1	0	0	0	0&1	0	0	0
0	?	0	0	0	0	0	0	1
0	1	0	0	0	0	0	0	0
0	1	0	0	1	1	1	0	0
0	?	0	0	1	1	0	0	0
0	1	0	0	0	1	0	0	0
1	?	?	0	0	1	0	0	0
1	1	0	0	0	1	0	0	1
0	1	1	0	0	1	0	0	0
0	1	1	0	0	1	0	0	0
0	?	0	0	0	1	0	0	1
0	1	0	0	0	1	0	0	1
0	?	?	0	0	1	0	0	0
1	1	0	0	0	1	0	0	0
0	1	0	0	0	1	0	0	0
0	?	0	1	0	1	0	0	0
0	1	0	0	0	1	0	1	0
0	?	?	1	0	1	0	0	0
0	1	?	1	0	1	1	0	0
0	?	?	1	0	1	0	0	0
1	1	?	0	0	1	0	0	0
1	1	0	0&1	0	1	0	0	0
0	?	0	0	0	1	0	0	0
0	?	0	0	0	1	0	0	0
?	1	0	0	0	1	0	0	0
1	1	0	0	0	1	1	0	0
1	0	0	0	0	1	1	0	0
1	0	0	0	0	1	1	0	0
0	0	?	0	0	1	0	0	0
0	0	0	0	0	1	0	0	0
1	1	?	0	0	1	0	0	1
0	1	0	0	0	1	1	0	1
0	1	?	0	0	1	1	0	0
1	1	0	0	0	1	1	0	1
1	1	?	0	0	1	0	0	0
1	1	0	0	0	1	1	0	0
1	0	0	0	0	1	0	0	0
?	?	?	?	?	?	?	1	?
?	?	?	?	?	?	?	1	?
0	1	0	0	0	1	1	1	1
1	0	0	0&1	0	1	1	0	0
0	1	?	0	0	1	1	1	1
?	?	?	?	?	?	1	0	1

153
0
0
0
0
0
0
0
0
0
0
0&1
0
0
0
0
0
0
0
0
0
0
0
0
0
0
0
0
0
0
0&1
0
0
0
0
0&1
0
0
0
0&1
0&1
?
?
1
0
0
1
