# Supplementary material for: A North American stem turaco, and the complex biogeographic history of modern birds
Source: BMC Evol Biol. 2018 Jun 25;18:102. doi: 10.1186/s12862-018-1212-3 (PMC6016133; doi:10.1186/s12862-018-1212-3)
Supplement: Supplementary file 4 — Table S2. Hindlimb measurements for fossil and recent Otidimorphae. (PDF 70 kb) [file 12862_2018_1212_MOESM4_ESM.pdf]

Species	Family	Sex	Museum specimen number	Femur Length (mm)	Tibiotarsus Length (mm)	Tarsometatarsus Length (mm)	% Femur of total hindlimb length	% Tibiotarsus of total hindlimb length	% Tarsometatarsus of total hindlimb length	Tarsometatarsus + Tibiotarsus (mm)	Hindlimb Index
Tauraco corythaix	Musophagidae	M	YPM 105024	56.43	77.67	45.02	31.50	43.36	25.13	122.69	2.17
Tauraco schalowi	Musophagidae	F	YPM 14925	52.13	72.04	40.28	31.70	43.81	24.49	112.32	2.15
Musophaga violacea	Musophagidae	F	YPM 142805	56.31	78.63	44.39	31.40	43.85	24.75	123.02	2.18
Corythaeola cristata	Musophagidae	F	YPM 142738	79.16	113.54	59.46	31.39	45.03	23.58	173.00	2.19
Corythaixoides leucogaster	Musophagidae	F	YPM 84711	49.32	72.83	42.55	29.95	44.22	25.83	115.38	2.34
Corythaixoides concolor	Musophagidae	M	YPM 105927	49.06	71.71	40.07	30.50	44.58	24.91	111.78	2.28
Geococcyx californianus	Cuculidae	?	YPM 5920	54.94	85.20	64.08	26.90	41.72	31.38	149.28	2.72
Geococcyx velox	Cuculidae	M	YPM 105878	49.72	72.77	53.54	28.25	41.34	30.42	126.31	2.54
Guira guira	Cuculidae	F	YPM 84673	37.27	57.61	40.76	27.48	42.47	30.05	98.37	2.64
Tapera naevia	Cuculidae	?	YPM 137581	27.47	41.59	31.73	27.25	41.26	31.48	73.32	2.67
Dromococcyx phasianellus	Cuculidae	M	YPM 144530	31.80	50.24	38.51	26.38	41.68	31.95	88.75	2.79
Crotophaga major	Cuculidae	?	YPM 102338	41.11	65.95	43.89	27.23	43.69	29.08	109.84	2.67
Crotophaga ani	Cuculidae	M	YPM 103238	35.90	57.49	38.94	27.13	43.44	29.43	96.43	2.69
Crotophaga sulcirostris	Cuculidae	F	YPM 105873	31.51	50.39	33.31	27.35	43.74	28.91	83.70	2.66
Saurothera longirostris	Cuculidae	M	YPM 105223	38.83	55.39	37.36	29.51	42.10	28.39	92.75	2.39
Piaya minuta	Cuculidae	F	YPM 105732	25.89	38.42	26.84	28.40	42.15	29.45	65.26	2.52
Piaya cayana	Cuculidae	F	YPM 105897	41.60	59.30	40.72	29.37	41.87	28.75	100.02	2.40
Coccyzus americanus	Cuculidae	?	YPM 102447	27.90	37.96	25.48	30.55	41.56	27.90	63.44	2.27
Coccyzus melacoryphus	Cuculidae	?	YPM 101048	28.26	40.10	26.09	29.92	42.46	27.62	66.19	2.34
Coccyzus erythropthalmus	Cuculidae	F	YPM 103278	26.76	37.85	24.31	30.09	42.57	27.34	62.16	2.32
Coccycua minuta	Cuculidae	?	YPM 137255	25.92	38.50	27.39	28.23	41.93	29.83	65.89	2.54
Coua cristata	Cuculidae	F	YPM 84663	36.08	59.50	41.62	26.30	43.37	30.34	101.12	2.80
Cacomantis flabelliformis	Cuculidae	?	YPM 137667	22.17	30.63	20.15	30.39	41.99	27.62	50.78	2.29
Cacomantis castaneiventris	Cuculidae	?	YPM 106084	19.13	27.66	18.47	29.31	42.38	28.30	46.13	2.41
Cuculus canorus	Cuculidae	F	YPM 105038	28.69	39.12	22.55	31.75	43.29	24.96	61.67	2.15
Chrysococcyx caprius	Cuculidae	M	YPM 105031	19.11	27.16	17.41	30.01	42.65	27.34	44.57	2.33
Chrysococcyx minutillus	Cuculidae	F	YPM 105428	18.05	26.78	17.75	28.84	42.79	28.36	44.53	2.47
Chrysococcyx lucidus	Cuculidae	M	YPM 110757	18.70	27.08	17.98	29.33	42.47	28.20	45.06	2.41
Chrysococcyx klaas	Cuculidae	M	YPM 103529	17.62	25.15	15.62	30.18	43.07	26.75	40.77	2.31
Centropus phasianinus	Cuculidae	?	YPM 105532	57.21	78.85	55.31	29.89	41.20	28.90	134.16	2.35
Centropus monachus	Cuculidae	M	YPM 107062	49.80	70.55	49.22	29.37	41.61	29.03	119.77	2.41
Centropus superciliosus	Cuculidae	F	YPM 105285	42.62	59.55	40.17	29.94	41.84	28.22	99.72	2.34
Ceuthmochares aereus	Cuculidae	F	YPM 107064	31.60	43.72	28.54	30.43	42.10	27.48	72.26	2.29
Phaenicophaeus calyorhynchus	Cuculidae	?	YPM 102335	46.19	65.59	43.31	29.78	42.29	27.93	108.90	2.36
Tetrax tetrax	Otididae	?	YPM 102062	59.43	97.31	65.14	26.78	43.86	29.36	162.45	2.73
Ardeotis arabs	Otididae	?	YPM 102540	112.44	233.80	186.10	21.12	43.92	34.96	419.90	3.73
Otis tarda	Otididae	M	YPM 102227	126.46	216.80	153.20	25.47	43.67	30.86	370.00	2.93
Ardeotis kori	Otididae	?	YPM 142264	121.06	246.20	194.90	21.53	43.80	34.67	441.10	3.64
Foro panarium	Foratidae	?	USNM 336261	54.10	88.40	61.30	26.55	43.38	30.08	149.70	2.77
Eocuculus cf. cherpinae	Eocuculidae	?	SMF Av 425	25.10	35.35	17.65	32.14	45.26	22.60	53.00	2.11
