# Supplementary figures and images for: A North American stem turaco, and the complex biogeographic history of modern birds
Source: BMC Evol Biol. 2018 Jun 25;18:102. doi: 10.1186/s12862-018-1212-3 (PMC6016133; doi:10.1186/s12862-018-1212-3)

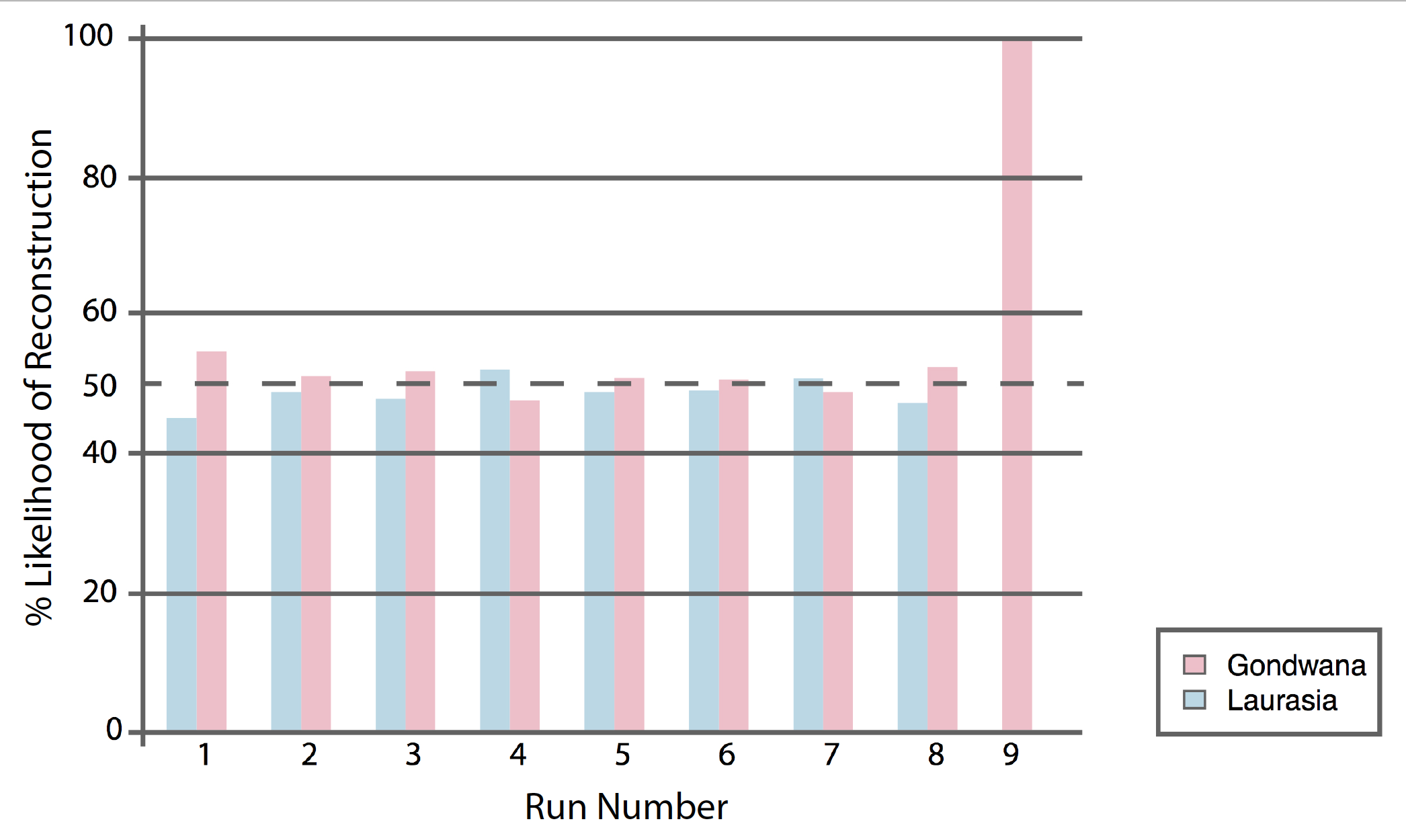

Supplement: Supplementary file 5 — Figure S1. Relative likelihood of a Gondwanan vs. Laurasian avian common ancestor based on alternative parameterization of the historical biogeographic analyses. (PNG 211 kb) [file 12862_2018_1212_MOESM5_ESM.png]
